# The Role of Nucleosome Positioning in the Evolution of Gene Regulation

**DOI:** 10.1371/journal.pbio.1000414

**Published:** 2010-07-06

**Authors:** Alexander M. Tsankov, Dawn Anne Thompson, Amanda Socha, Aviv Regev, Oliver J. Rando

**Affiliations:** 1 Broad Institute of MIT and Harvard, Cambridge, Massachusetts, United States of America; 2 Department of Electrical Engineering and Computer Science, Massachusetts Institute of Technology, Cambridge, Massachusetts, United States of America; 3 Department of Biology, Massachusetts Institute of Technology, Cambridge, Massachusetts, United States of America; 4 Howard Hughes Medical Institute, Cambridge, Massachusetts, United States of America; 5 Department of Biochemistry and Molecular Pharmacology, University of Massachusetts Medical School, Worcester, Massachusetts, United States of America; Adolf Butenandt Institute, Germany

## Abstract

Chromatin organization plays a major role in gene regulation and can affect the function and evolution of new transcriptional programs. However, it can be difficult to decipher the basis of changes in chromatin organization and their functional effect on gene expression. Here, we present a large-scale comparative genomic analysis of the relationship between chromatin organization and gene expression, by measuring mRNA abundance and nucleosome positions genome-wide in 12 *Hemiascomycota* yeast species. We found substantial conservation of global and functional chromatin organization in all species, including prominent nucleosome-free regions (NFRs) at gene promoters, and distinct chromatin architecture in growth and stress genes. Chromatin organization has also substantially diverged in both global quantitative features, such as spacing between adjacent nucleosomes, and in functional groups of genes. Expression levels, intrinsic anti-nucleosomal sequences, and *trans*-acting chromatin modifiers all play important, complementary, and evolvable roles in determining NFRs. We identify five mechanisms that couple chromatin organization to evolution of gene regulation and have contributed to the evolution of respiro-fermentation and other key systems, including (1) compensatory evolution of alternative modifiers associated with conserved chromatin organization, (2) a gradual transition from constitutive to *trans*-regulated NFRs, (3) a loss of intrinsic anti-nucleosomal sequences accompanying changes in chromatin organization and gene expression, (4) re-positioning of motifs from NFRs to nucleosome-occluded regions, and (5) the expanded use of NFRs by paralogous activator-repressor pairs. Our study sheds light on the molecular basis of chromatin organization, and on the role of chromatin organization in the evolution of gene regulation.

## Abstract 2

A comparative genomics study maps nucleosomes across the entire genomes of 12 fungal species, identifying multiple distinct mechanisms linking changes in chromatin architecture to evolution of gene regulation.

## Author Summary

Divergence in gene regulation plays a major role in organismal evolution. Evidence suggests that changes in the packaging of eukaryotic genomes into chromatin can underlie the evolution of divergent gene expression patterns. Here, we explore the role of chromatin structure in regulatory evolution by whole-genome measurements of nucleosome positions and mRNA levels in 12 yeast species spanning ∼250 million years of evolution. We find several distinct ways in which changes in chromatin structure are associated with changes in gene expression. These include changes in promoter accessibility, changes in promoter chromatin architecture, and changes in the accessibility of specific transcription factor binding sites. In many cases, changes in chromatin architecture are coupled to physiological diversity, including the evolution of a respiration- or fermentation-based lifestyle, mating behavior, salt tolerance, and broad aspects of genomic structure. Together, our data will provide a rich resource for future investigations into the interplay between chromatin structure, gene regulation, and evolution.

## Introduction

Regulatory differences affecting gene expression can play a major role in species evolution [1] and can help elucidate the functional mechanisms that control gene regulation [2],[3]. Although specific examples of regulatory divergence are known in bacteria [4], fungi [5],, flies [9], and mammals [10], a general understanding of the evolution of gene regulation is still lacking. The recent availability of many sequenced genomes and accessibility of genomic profiling approaches open the way for comparisons of gene regulation across multiple species.

**Figure 1:**
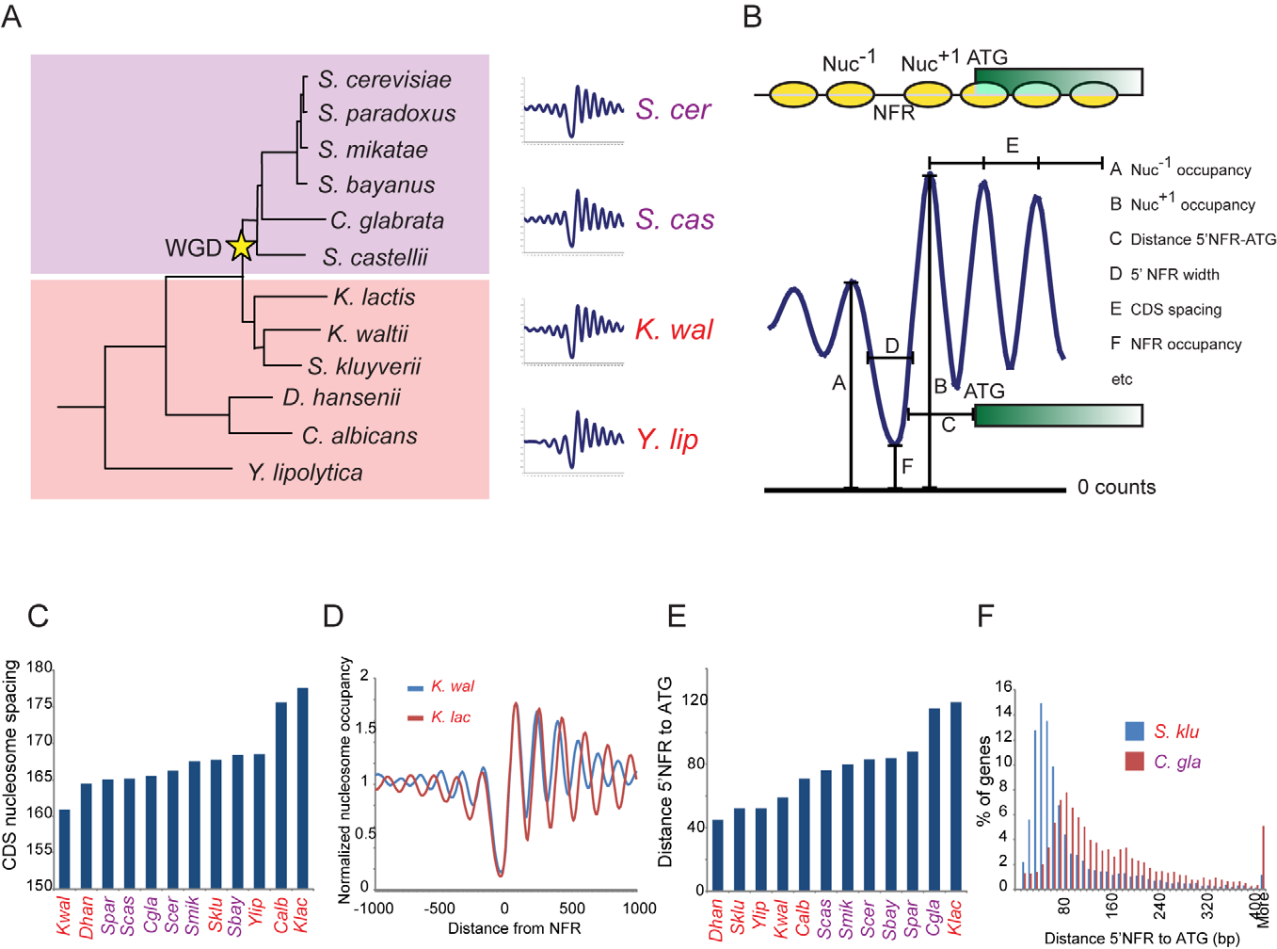
Global chromatin organization in 12 *Hemiascomycota* fungi. (A) Phylogeny of species included in this study (adapted from [34]). Right, gene-averaged nucleosome sequencing data from 4 of the 12 species, aligned by *Nuc^+1^*. (Data for all species are in Figure S2.) (B) Chromatin features. Shown is a schematic of a gene (green box), its promoter (black line) and associated nucleosomes (yellow), along with nucleosome sequencing data (dark blue curve), and several extracted features, as indicated. (C) Global variation between species in nucleosome spacing in coding regions. Shown are the median nucleosome-to-nucleosome distances over coding regions, averaged over all genes in each species. Values are arranged from low to high rather than by phylogeny to emphasize the range of variability. Species names are colored by their relation to WGD as in (A). (D) Spacing differences between two *Kluyveromyces* species. Shown are 5′ NFR-aligned averaged data for *K. lactis* (red) and *K. waltii* (blue), showing differences in coding region spacing. (E) Global variation in NFR to ATG distance (*D_5′NFR-ATG_*). Shown are median distances from the 5′ NFR to start codon for all genes in each species, sorted from low to high values. (F) Distribution of NFR to ATG distances (*D_5′NFR-ATG_*) in *S. kluyverii* (blue) and *C. glabrata* (red).

Among eukaryotes, the *Hemiascomycota* yeasts (Figure 1A), which span over ∼250 million years of evolution, are particularly suitable for studying evolution of gene regulation. This is due to the genetic tractability of yeasts, the wealth of knowledge about the model organism *Saccharomyces cerevisiae*, the large number of sequenced genomes, and the diversity of yeast lifestyles [3]. Notably, *Hemiascomycota* yeasts diverged before and after a whole genome duplication event (WGD, Figure 1A) [11], which marked a shift from using respiration for energy production in pre-WGD species to primarily using fermentation in post-WGD species [12].

Nucleosomes modulate eukaryotic gene regulation by affecting the accessibility of other proteins to the DNA, which can impact gene activation and repression [13]. In particular, many genes have nucleosome-depleted “Nucleosome Free Regions” (NFRs) in their proximal promoters (Figure 1B, top), providing access to sequence specific transcription factors (TFs) and to the basal transcription machinery [14],[15],[16],[17]. Three major determinants have been proposed to impact nucleosome depletion at NFRs: (1) active transcription by RNA polymerase II results in eviction of the −1 nucleosome [18],[19], (2) intrinsic “anti-nucleosomal” DNA sequences such as Poly(dA:dT) bind histones with low affinity and can “program” NFRs constitutively [20],[21],[22],[23],[24], and (3) *trans*-acting proteins can move nucleosomes away from their thermodynamically preferred locations [25],[26].

Recent studies in yeast suggest a broad role for chromatin organization in regulatory evolution. Most regulatory divergence between closely related *S. cerevisiae* strains is associated with divergence in unlinked (*trans*) chromatin remodelers [27],[28]. Conversely, many transcriptional differences between *S. cerevisiae* and *S. paradoxus* (Last Common Ancestor (LCA) ∼2 million years ago (MYA)) are due to linked *cis* polymorphisms predicted to affect nucleosome occupancy [29],[30]. Furthermore, a recent study suggested that changes in the regulation of mitochondrial ribosomal protein (mRP) genes between the distant species *C. albicans* and *S. cerevisiae* (LCA ∼200 MYA) were associated with a change in nucleosome organization [31],[32]. In particular, the higher expression of mitochondrial genes in respiratory *C. albicans* is accompanied by enrichment for the PolyA-like “RGE” binding site in the mRP gene promoters [31], which appears to “program” the constitutive presence of wider, more open NFRs at these genes [32]. All of these are absent from the promoters of mRPs in the fermentative *S. cerevisiae*. Finally, a recent study [33] compared genome-wide nucleosome positioning in *S. cerevisiae* and *S. pombe* (LCA ∼300M–1 BYa), finding changes in global nucleosome spacing and in the apparent sequences that intrinsically contribute to nucleosome positioning in vivo.

While these examples are intriguing, they are limited in their phylogenetic coverage (a pair of species) or their functional scope (one regulon). Thus, we understand little about the evolutionary interplay between gene expression, regulatory sequence elements, and chromatin organization. How does chromatin organization change over evolutionary time scales? Are the mechanisms underlying chromatin packaging of functional gene modules conserved? If not, how do they evolve and what is the role of different factors in this divergence? Are changes in chromatin organization related to changes in gene regulation? Can phylogenetic comparisons shed light on the distinct mechanisms that help establish chromatin organization?

Here, we present the first large-scale experimental and computational study of chromatin organization across a eukaryotic phylogeny. We measured genome-wide nucleosome locations and mRNA abundance in 12 *Hemiascomycota* yeast species, spanning over 250 million years of evolution (Figure 1A). We developed an analysis framework that integrates the experimental data with genome sequences, functional gene sets, and TF binding sites across the 12 species.

Our analysis uncovers several major principles that govern the evolutionary and functional relationship between chromatin organization and gene regulation in this phylogeny. (1) While qualitative features of chromatin organization are conserved in all species, quantitative features such as nucleosome packing, NFR length, and NFR to ATG distance have substantially diverged; (2) promoter chromatin organization and gene expression levels of “growth” and “stress” genes follow distinct patterns, and this dichotomy is conserved in all species; (3) evolutionary divergence in gene expression is often accompanied by transition of chromatin organization from a “growth” to a “stress” pattern; (4) changes in transcription levels, gain/loss of anti-nucleosomal sequences, and gain/loss of binding sites for “general regulatory factors” (GRFs) all play substantial and complementary roles in divergence of chromatin organization; (5) the loss of anti-nucleosomal sequences and parallel gain of binding sites for GRFs drive shifts from intrinsic to *trans*-regulated chromatin organization; (6) regulatory divergence can also occur by re-positioning of binding sites relative to nucleosome positions or by expanding the use of accessible sites by paralogous TFs. These mechanisms played a role in the evolution of respiro-fermentation, as well as in the evolution of regulation of other key regulons at different phylogenetic points, including mating, meiosis, RNA polymerase subunits, proteasomal, and splicing genes. Together, they uncover novel insights into the general roles for chromatin in regulating genomic access and in the evolution of regulatory programs, and provide a rich resource for future investigation.

## Results

### A Chromatin Map for 12 *Hemiascomycota* Species

We mapped nucleosome positions genome-wide in 12 *Hemiascomycota* species (Figure 1A) [34] by Illumina sequencing of mononucleosomal DNA [19],[21],[35] isolated from mid-log cultures (Materials and Methods, Figures 1A and S1). To minimize condition- and stress-related differences, we grew all species in the same rich medium, where the growth rate of each species was at least ∼80% of its maximal measured rate in any of over 40 tested media formulations. In order to compare our data to transcriptional output, we also used species-specific microarrays to measure mRNA abundance in all species in the same mid-log cultures used for nucleosome mapping (Table S2, Materials and Methods).

Aligning nucleosome reads to each genome and averaging over all genes showed remarkably similar profiles in all species studied (Figures 1A, S2, S3). All gene-averaged profiles are dominated by a pronounced depression upstream of the ATG that corresponds to the NFR [14],[15],[16],[17],[36]. To quantitatively compare chromatin structure between various genes, we first called nucleosome positions, identified 5′ and 3′ NFRs, and measured a number of nonredundant features that describe the chromatin organization at each gene (Materials and Methods, Figures 1B and S4). Below, we will study each feature at three levels: (1) *globally*, averaged across all genes in a genome; (2) *functionally*, averaged across all genes in a functional category; and (3) *locally*, at a single gene.

### Packaging of Coding Regions Is Qualitatively Conserved, but Quantitative Features Such as Nucleosome Spacing and NFR Width Have Diverged between Species

Several qualitative chromatin features have previously been identified in all eukaryotes studied [14], and these are conserved across all 12 species (Figures 1A, S2, and S3). These include an abundant 5′NFR, a common 3′NFR, a well-positioned +1 nucleosome (Nuc^+1^), and increasing nucleosome fuzziness over the body of genes (Figures S2 and S3, Table S3), which is consistent with statistical positioning of nucleosomes [23],[37],[38].

In contrast, quantitative global features were often variable between species (Figures 1C–F and S5, Table S3). Our measurements recapitulated previous predictions or bulk assays in the few cases where these were available, thus validating our dataset and analytical methods. For example, nucleosome spacing in coding regions was variable between species (Figure 1C,D), consistent with observed nucleosome laddering on gels [39],[40]. This leads to variation in the specific coding sequences exposed in linker DNA and could affect patterns of sequence variation [41],[42],[43] and higher-order packaging into the 30 nm fiber [44]. The distance between the NFR and a gene's start codon (Figures 1E,F and S5) is also variable between species, consistent with prior computational predictions [45].

Other evolutionary variations in global features were not previously described, showing that additional major aspects of chromatin architecture can substantially diverge. Most notably, the median NFR width was highly variable between species (Table S3), ranging from 109 to 155 nucleotides. This likely reflects the variation in the length and abundance of anti-nucleosomal Poly(dA:dT) tracts between species (discussed below). Shorter NFRs may constrain regulatory information into more compact promoters.

### A Conserved Dichotomy in Chromatin Organization of “Stress” and “Growth” Genes

We next explored possible functional implications of chromatin organization in specific sets of genes with related function. Prior studies in *S. cerevisiae* and *C. albicans* have shown that in both species, “growth” genes, defined by their co-expression with cytoplasmic ribosomal proteins (cRPs), have a more open chromatin organization on average [32]. Conversely, “stress” genes, whose expression is anti-correlated to that of growth genes, have a more closed chromatin organization in both species.

**Figure 2:**
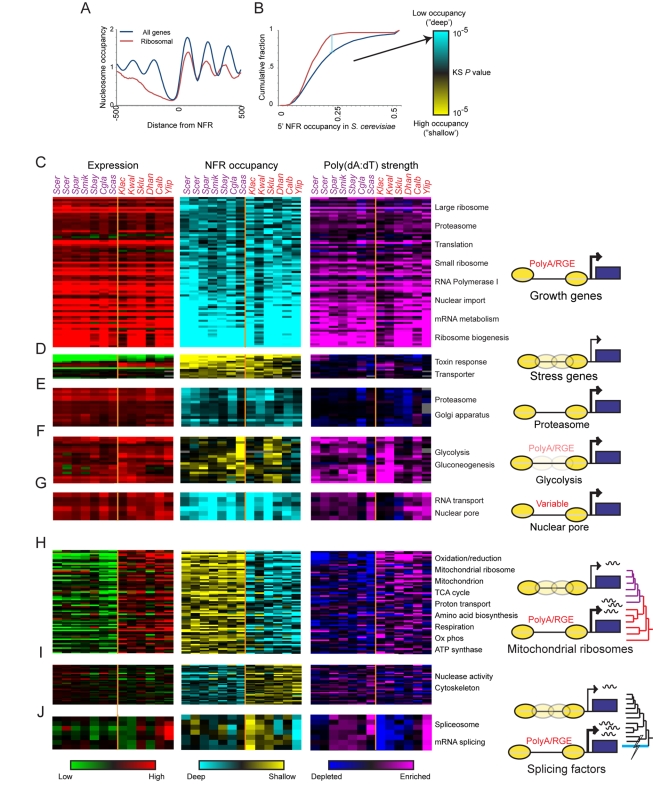
Conservation and variation in chromatin structure of functional gene sets. (A,B). Strategy for associating chromatin features with gene sets. (A) Shown is *Nuc^+1^*-aligned nucleosome data for all genes (blue) and ribosomal protein genes (red) in *S. cerevisiae*, demonstrating that ribosomal protein genes are associated with wider NFRs. (B) Cumulative distribution plot of NFR occupancy in all genes (blue) *versus* ribosomal protein genes (red). *y*-axis shows fraction of promoters with NFR occupancy below a given value, with NFR occupancy values on the *x*-axis. Wide separation between curves (light blue vertical line) is captured by a significant K-S statistic, indicating that ribosomal genes have significantly low occupancy, or “deep” NFRs. K-S *P* values are converted to color scale (right panel): blue, significantly low feature values; yellow, significantly high feature values. (C–J) Conservation and variation in chromatin organization in specific gene sets. Shown are the K-S statistics for expression level (red, high expression; green, low expression; left panel), NFR occupancy (yellow/blue, middle panel), and Poly(dA:dT) tracts in NFRs (purple, high Poly(dA:dT) strength enrichment; dark blue, low strength enrichment; right panel) for gene sets (rows) with distinct phylogenetic patterns across the 12 species (columns; species names are color coded by WGD). K-S *P* values at saturation are 10^−20^ (Expression, C–E), 10^−5^ (occupancy and PolyA, C–E), 10^−10^ (Expression, F–G), 10^−2.5^ (occupancy and PolyA F–G). For (H–J), all gene sets are normalized to an average row value of zero (i.e., centered to show relative changes), and *P* value saturation values are 10^−8^ (expression) and 10^−2^ (occupancy, PolyA). Also shown are cartoons (right) reflecting the chromatin organization inferred from the test and relevant phylogenetic events. (C) Conserved deep NFRs in growth genes, associated with high expression and strong Poly(dA:dT) tracts; (D) conserved occupied NFRs in stress genes, associated with low expression and weak Poly(dA:dT) tracts; (E) conserved deep NFRs in proteasome genes associated with high expression but not with Poly(dA:dT) tracts; (F) conserved occupied NFRs in glycolysis genes despite high expression; (G) deep NFRs and high expression at nuclear pore genes associated with Poly(dA:dT) tracts only in a subset of species; (H) divergence from deep to occupied NFRs following the WGD at mitochondrial protein genes, associated with reduction in expression and in Poly(dA:dT) tracts; (I) divergence from occupied to deep NFRs following the WGD in cytoskeletal genes, despite little change in expression or Poly(dA:dT) tracts; (J) divergence from deep to occupied NFRs in splicing after the divergence of *Y. lipolytica* associated with reduction in expression and in poly dA:dT tracts.

To assess the generality of this observation, and identify additional trends, we tested in each species thousands of functional gene sets for enrichment of each of 22 distinct chromatin parameters. We used gene orthology [34] to project functional gene sets defined in *S. cerevisiae* across species (Materials and Methods). For a given gene set in each species we calculated whether its constituent genes tended to have high or low values of each of the chromatin features (Figure 1B), relative to the background of that feature's overall distribution in that species (Kolmogorov-Smirnov (K-S) test, Figure 2A,B). This provides a comprehensive overview of chromatin organization at 5′ promoters and 3′ ends for each functional gene set across the 12 species (Figure 2C–J, middle panels, Figures S6 and S7, and Tables S4–S5). In order to compare chromatin changes to gene expression levels, we also calculated the enrichment for high or low mRNA expression in all gene sets for each species (K-S test, Figure 2C–J, left panels).

We confirm a strong dichotomy in the promoter chromatin architecture of most “stress” and “growth” genes in *S. cerevisiae* [19],[46],[47],[48],[49] and *C. albicans* [32] and find that it is conserved across all 12 species (Figures 2C,D and S6–S8). Promoters of “growth” genes (e.g., ribosomal, proteasomal, and nuclear pore proteins, Figure 2C,E,G) exhibit long and deep (low occupancy) 5′NFRs. Conversely, those of “stress” genes (e.g., toxin-response genes, integral membrane proteins, Figure 2D) exhibit a more variable chromatin architecture, with shallower (higher occupancy) and narrower 5′NFRs. A host of other chromatin features also distinguish between the two functional groups (Figure S6). Thus, the separation of the “growth” and “stress” axes is a hallmark of *Hemiascomycota* gene regulation [2],[3] and imposes strong constraints at all levels from evolution of gene content [34] to chromatin organization. There are, however, several exceptions to this rule. Most notably, several key “growth” genes, including glycolysis genes and endoplasmic reticulum genes, are highly expressed, yet do not exhibit deep NFRs in any species (Figure 2F).

We identify a range of additional conserved patterns of chromatin architecture associated with other specific functions, which were not previously reported. For example, a number of gene sets (e.g., reproduction, cell wall, inositol phosphate, benzoate, and nicotinamide metabolism genes) have conserved long NFR to ATG distances (Figure S6), but have few other hallmarks of stress genes, and are expressed at average levels. In *S. cerevisiae*, these genes have long 5′ untranslated regions (5′UTRs) [50], suggesting that relatively long 5′UTRs are conserved at their orthologs in all 12 species. This may indicate a conserved role for translational control in the regulation of these functions [51].

### Coherent Changes in Chromatin Organization Accompanied the Evolutionary Divergence of Gene Regulation in Mitochondrial, Splicing, and Cytoskeleton Genes

On this backdrop of conservation, we find that coordinated changes have occurred in chromatin organization of specific functional gene sets, consistent with major phenotypic changes. Most notably, respiration and mitochondrial genes have switched from a “growth”-like chromatin pattern in pre-WGD species (where they are highly expressed) to a more “stress”-like pattern post-WGD (Figures 2H and S6). We confirm the previously reported change between *S. cerevisiae* and *C. albicans* for genes involved in respiratory metabolism [32]. We further extend these results across the full phylogenetic scope and to several other gene sets of related function (Figures 2H and S6). This change corresponds to a major change in lifestyle from respiration to respiro-fermentation after the WGD [12],[31],[32],[52]. We also discover the converse evolutionary pattern (Figure 2I): a number of gene sets involved in cytoskeletal organization are packaged into deeper NFRs in post-WGD species than in pre-WGD species. Surprisingly, the expression level of these genes has not substantially changed with this transition.

Changes in chromatin organization have also occurred at other phylogenetic points of phenotypic evolution, suggesting a general evolutionary mechanism. For example, we discovered that in *Yarrowia lipolytica* spliceosome genes are associated with long and deep NFRs, but in all other species they are enriched for short and shallow NFRs (Figure 2J, middle panel). This switch between deep and shallow NFRs is accompanied by a decrease in expression of these genes (Figure 2J, left panel) and is consistent with the much larger number of introns in *Yarrowia lipolytica* genes [53] and with the loss of introns and reduction of splicing in the subsequently diverged species.

### Differences in Expression and Intrinsic Anti-Nucleosomal Sequences Only Account for Some of the Changes in Chromatin Organization Within and Between Species

We next asked what mechanisms contribute to conservation and variation in chromatin organization across species. Three determinants have been previously implicated in establishing NFRs in *S. cerevisiae* [14]: (1) the expression level of the gene, as RNA polymerase recruitment affects NFR width; (2) the presence of intrinsic anti-nucleosomal sequences such as Poly(dA:dT) tracts in the gene's promoter; and (3) the binding of proteins such as chromatin remodelers that actively evict or move nucleosomes. We first consider these three determinants independently, and then assess their relative contributions.

In some cases, variation in chromatin organization in a gene set, both within and between species, correlates with gene expression level. Within each species, many highly expressed “growth” genes (e.g., RP genes) are packaged with wide and deep NFRs, while many poorly expressed stress genes have shorter, occupied NFRs (Figures 2C,D, S6). Between species, evolutionary shifts from high to low expression levels were sometimes accompanied by corresponding changes in chromatin organization (e.g., mitochondrial RP and splicing genes, Figure 2H,J).

However, transcription level is insufficient to solely explain the NFR occupancy measured across the 12 species. Globally, expression level alone explains only 1.7%–13.1% of the variation in NFR occupancy in each of the 12 species (Lowess fit, Figure S9A,C,E, Materials and Methods). Furthermore, when we use Lowess subtraction to correct for the relationship between mRNA level and each chromatin feature, the enrichments of most gene sets for high or low values of chromatin features were maintained (Figure S10, Materials and Methods). Within species, the discrepancy is prominent in some of the gene sets (e.g., glycolysis, gluconeogenesis) that are highly expressed in all species but do not exhibit the expected deep NFRs (Figure 2F). Between species, cytoskeleton and nuclease-related gene sets have shifted from shallow to deep NFRs at the WGD, often without a concomittant change in expression levels (Figure 2I). The failure of transcript levels to fully explain NFR width and depth is consistent with recent experimental results in *S. cerevisiae*, where the distinctive chromatin organization of growth and stress genes was largely maintained even after genetically inactivating RNA Pol II [19].

We next tested an alternative hypothesis that chromatin organization at the NFR is determined by intrinsic “anti-nucleosomal” sequences with low affinity for the histone octamer, such as Poly(dA:dT) tracts [20],[21],[22],[24],[54],[55]. We estimated the average extent of nucleosome depletion over a variety of Poly(dA:dT) elements (Materials and Methods) for each species (Figures S11, S12). We then tested if functional gene sets in each species were enriched or depleted for strongly anti-nucleosomal sequences in their NFRs. Finally, we compared this pattern to their chromatin organization (Figure 2C–J, right versus middle panels).

In some cases, the variation in chromatin organization within and between species is associated with variation in intrinsic “anti-nucleosomal” Poly(dA:dT) tracts. Within each species, Poly(dA:dT) sequences are enriched upstream of many highly expressed, nucleosome-depleted, “growth” gene sets, consistent with previous observations in *S. cerevisiae* [48],[49]. Between species, we found that gain and loss of polyA sequences is associated with changes in chromatin organization at several gene sets and phylogenetic points, suggesting that this is a common evolutionary mechanism used more than once in this phylogeny. We confirmed a prior observation [32] that the change in chromatin organization at mitochondrial ribosomal protein (mRP) genes in post-WGD respiro-fermentative species is accompanied by the loss of PolyA-like sequences from these promoters (Figure 2H). In addition, we found that the deeper and wider NFRs at splicing genes in *Y. lipolytica* are associated with a greater length and number of PolyA sequences at these genes (Figure 2J). Conversely, the relatively shallow NFRs of gluconeogenesis genes observed in *S. castellii* are associated with concomitant depletion of polyA sequences in this species (Figure 2F).

Nevertheless, intrinsic anti-nucleosomal sequences explain only 8.6%–25.7% of the global variation in NFR occupancy within a given species (Figure S9). Even when combining expression levels and sequence information together, these can only explain 13%–29% of the global variation in nucleosome organization in the 12 species (Figure S9E). Similar results are obtained when considering other measures of intrinsic anti-nucleosomal sequences, such as those based on computational models [21],[48] derived from in vitro data (unpublished analysis).

Thus, anti-nucleosomal sequences and expression patterns are insufficient to fully explain either conservation or divergence in chromatin organization across species. For example, proteasomal genes are highly expressed and have deep NFRs conserved in all species, but are not associated with intrinsic anti-nucleosomal sequences (Figure 2E). Furthermore, RNA Polymerase II subunits, RNA export, and nuclear pore genes are highly expressed with deep NFRs conserved in most species, but are enriched for intrinsically anti-nucleosomal sequences in only a subset of species (Figure 2G, see below). Conversely, peroxisome genes are highly expressed in *D. hansenii*, *C. albicans*, and *Y. lipolytica*, where they are packaged with long (but not deep) NFRs, despite no enrichment for Poly(dA:dT) tracts (see below). In these and other cases, even when we consider expression levels, much of the depletion in NFRs remained unexplained (Figures S9,S10).

### General Regulatory Factors (GRFs) Contribute to the Establishment of NFRs in Each Species

We therefore wished to explore the role that the third mechanism—nucleosome eviction by chromatin remodelers—plays across the 12 species. We hypothesized that changes in chromatin remodeling would be accompanied by variation in the *cis*-regulatory elements bound by GRFs that likely recruit chromatin remodelers [25],[56],[57]. Unlike intrinsic anti-nucleosomal sequences that establish constitutively programmed NFRs, binding sites for GRFs likely establish regulated NFRs that can change based on *trans* inputs.

We first assessed the potential contribution of chromatin remodelers to chromatin organization based on the presence in NFRs of the known binding sites for the two best-studied *S. cerevisiae* GRFs: Abf1 and Reb1 (Figure S9E, Materials and Methods). Together, the two motifs explain 1.2%–15.1% of the observed variation in nucleosome organization in the 12 species. Furthermore, Abf1 and Reb1 can explain up to 12.6% of the residual variation after accounting for the contribution of expression levels and intrinsic sequences (Successive Lowess, ). Thus, GRFs can play an important role in explaining global chromatin organization.

Notably, the Abf1 and Reb1 sites explain little of the variation in *D. hansenii*, *C. albicans*, and *Y. lipolytica*—the species from the two clades most distant from *S. cerevisiae*. In particular, the Abf1 binding site explains less than 1% of the variation in each of these species, consistent with the absence of the Abf1 ortholog from their genome, and validating the specificity of our approach. Furthermore, although the Reb1 ortholog is present in each of these species, its contribution is substantially reduced (compared to, e.g., *S. kluyveri*). This loss of predictive power by Abf1 and Reb1 sites at increasing phylogenetic distance led us to hypothesize that other GRFs, with distinct binding specificity, are active in these species.

**Figure 3:**
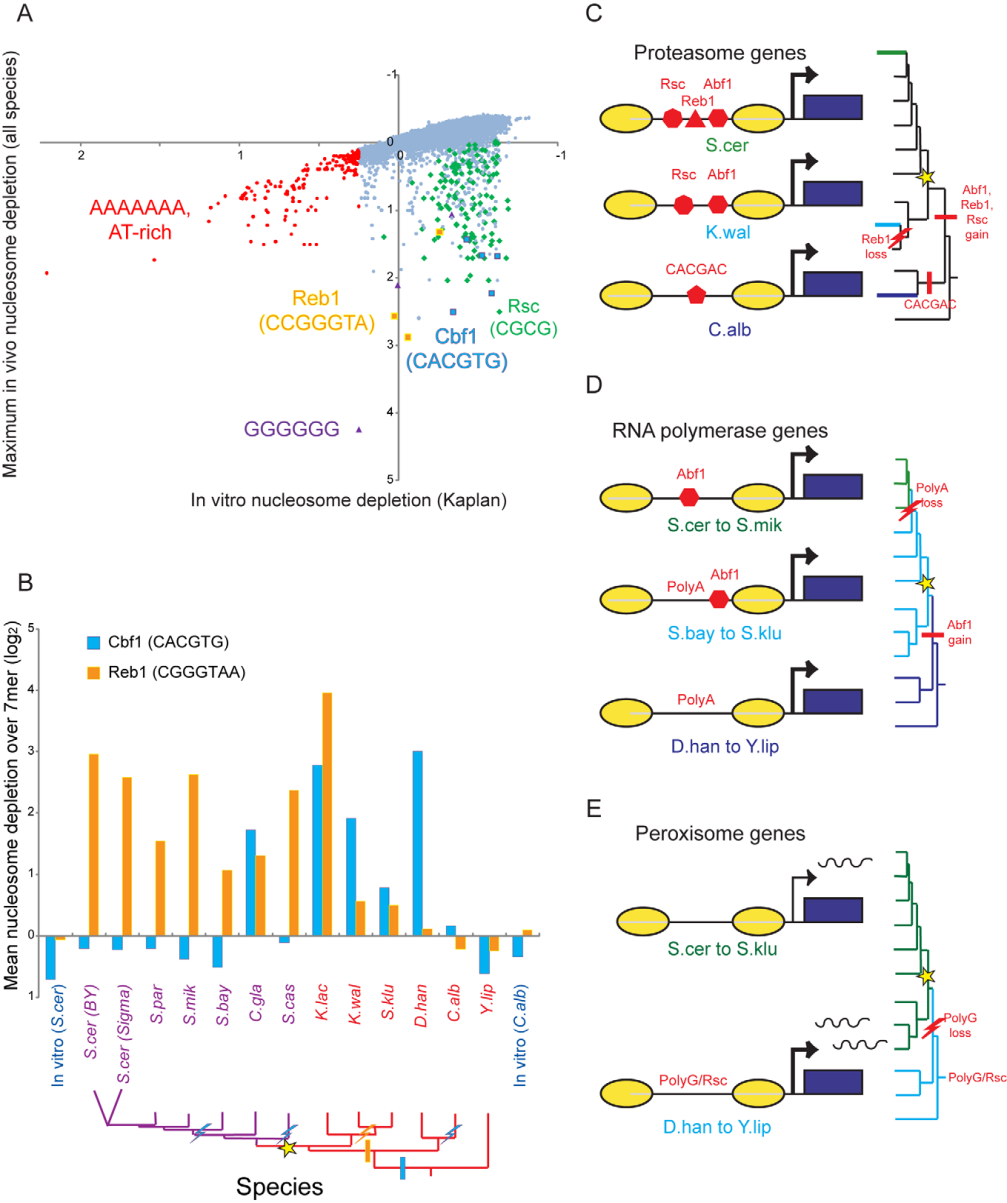
Evolution of sequence motifs associated with nucleosome depletion. (A) Identification of putative GRF sites. Nucleosome depletion scores were calculated over all 7-mers from in vitro reconstitution data [21] and from our in vivo data for all species (Materials and Methods). Scatter plot shows the in vitro depletion score (*x*-axis) versus the maximal 7-mer nucleosome depletion score observed in vivo in any of the 12 species (*y*-axis). Motifs corresponding to select known binding sites are indicated. (B) Evolutionary transition from the GRF Cbf1 to the GRF Reb1 through a redundant intermediate. Shown are the nucleosome depletion scores for the Cbf1 (blue) and Reb1 (orange) sites for the in vivo data from the 12 species (purple, red species as in Figure 1A), and for two published in vitro reconstitution datasets (blue) in *S. cerevisiae* [21] (left) and *C. albicans* (right) [32]. Bottom, phylogenetic tree marked with inferred events including the ancestral role of Cbf1 (blue bar), the gain of Reb1 (orange bar) and the loss of Cbf1's and Reb1's role as GRFs (lightning bolts). (C–E) Schematics of the evolution of usage of GRF and intrinsic anti-nucleosomal sites in proteasome genes (C), RNA polymerase genes (D), and peroxisome genes (E). Yellow ovals, nucleosomes; blue box, coding sequence; arrow, promoter; polyA, intrinsic anti-nucleosomal sequences; Abf1, Rsc, Reb1, CACGAC (*C.albicans*-specific GRF site), enriched GRF motifs. The phylogenetic tree is shown on the right, with the relevant clades in colors matching to the highlighted species. Bar, gain of functional site; lightning bolt, loss of functional site.

To identify novel GRF *cis*-elements, we therefore searched for short sequence elements that are depleted of nucleosomes in vivo but not in vitro [21]. We calculated the extent of nucleosome depletion over every 6- and 7-mer sequence in each of our species (Table S6, Materials and Methods) and identified those sequences whose depletion score in vivo in at least one species is significantly greater than expected from published in vitro data (Figure 3A, Figure S13–S14) [21]. This procedure automatically identified in vivo-specific depletion over 7-mers consistent with the binding sites for known *S. cerevisiae* GRFs such as Reb1 (Figure 3A, orange) [58],[59] and the Rsc3/30 components of the RSC ATP-dependent chromatin remodeling complex (Figure 3A, green) [25],[58],[59], validating our approach. Consistent with our hypothesis, it also revealed a number of sequence motifs that were specifically nucleosome-depleted in vivo in some species but not in *S. cerevisiae*, such as the CACGTG motif that serves as the binding site for Cbf1 in *S. cerevisiae* and *C. albicans* (Figure 3A, blue) [8],[58],[59],[60],[61]. We therefore propose that these sites are candidates for putative GRF function in these species.

### Phylogenetic Transitions in the Repertoire of GRFs That Recruit Chromatin Remodelers in *Hemiascomycota*

When we compared the GRF sequences between species we discovered extensive divergence that largely conforms to phylogenetic distance (Figures S13, S14). The extent of nucleosome depletion over short sequence elements is well conserved between closely related species, such as *S. cerevisiae* and *S. mikatae* (∼2–5 MYA, Figure S13B). In contrast, there are much more dramatic differences in the in vivo depleted sites (e.g., Rsc3/30, Cbf1) between the more distant *S. cerevisiae* and *K. lactis* (∼150 MYA, Figures S13C, S14). Finally, there are also gradual changes in the specific Rsc3/30 CGCG-containing motifs that were nucleosome-depleted in each species (Table S6), consistent with co-evolution of a GRF and its binding site, as previously observed for TFs [3],[5].

The use of different GRF sites often follows strong phylogenetic patterns, allowing us to trace transitions from the dominant use of one repertoire of GRFs to that of another, and suggesting compensatory evolution of GRF use. Most notably, we find a major and gradual transition from the use of Cbf1 as a major GRF in pre-WGD species to the use of Reb1 as a GRF in post-WGD species (Figure 3B). The Cbf1 binding sequence CACGTG is nucleosome-depleted in vivo in most pre-WGD species (except *Y. lipolytica* and *C. albicans*) but not in post-WGD species (except *C. glabrata*) (Figure 3B). Conversely, Reb1 sites are nucleosome depleted in all post-WGD species but not in most pre-WGD species (except *K. lactis*) (Figure 3B). This complementary phylogenetic pattern suggests an evolutionary scenario where Cbf1 was a major ancestral GRF, Reb1 emerged as a GRF before the WGD, and gradually “took over” Cbf1's global functionality. Similar evolutionary patterns were previously observed for TFs [3],[7],[61],[62], and this is the first demonstration to our knowledge of such a “mediated replacement” for GRFs. Evolutionary transitions in GRF usage are sometimes limited to one or a few species. For example, we found a set of novel motifs that were nucleosome-depleted only in *Y. lipolytica* (Table S6), the earliest diverging species in our panel.

Finally, we observe changes in the relative balance between nucleosome depletion via GRFs and constitutively programmed depletion via Poly(dA:dT) sequences, suggesting a global mode of compensatory evolution. Most notably, A7/T7 is less nucleosome-depleted at *D. hansenii* promoters than at promoters of any other species, whereas Cbf1-like and Rsc3/30-like sites are strongly nucleosome-depleted in *D. hansenii* (Figure S14). This transition is likely due to the shorter lengths of Poly(dA:dT) stretches in *D. hansenii* (Figure S11C, Table S7), a sequence change that may be an adaptation to the high salt concentrations in this species' ecological niche (secondary to increased DNA flexibility in high salt). As noted above, *D. hansenii* has a very short average NFR width (Table S3, Figure S11D), consistent with diminished nucleosome repulsion at its shorter Poly(dA:dT) sequences. We hypothesize that the expansion in use of the Cbf1 and Rsc3/30 GRFs is a mode of compensatory evolution needed to adapt to a change in genome sequence in a unique niche; it also suggests that *D. hansenii* NFRs may be more responsive to environmental signals.

### Divergent GRFs Underlie Conserved Chromatin Organization in Proteasome Genes

We next hypothesized that the identified GRFs are important for the observed chromatin organization in functional gene sets across species. To test this hypothesis, we assessed the enrichments of GRF motifs in the NFRs of each gene set across the 12 species (Table S8).

In some cases, GRF motifs (but not Poly(dA:dT) tracts) were enriched in a gene set across multiple species, strongly indicating a conserved regulatory mechanism. For example, the Abf1 site is enriched in RNA polymerase genes across the clade spanning *S. cerevisiae* and *S. kluyverii* (Figure S15D). However, since the spectrum of GRFs is species-specific (Figures 3B, S14), we found no gene set associated with the same GRF site across the entire phylogeny.

Instead, we found a number of cases where a single gene set has a conserved chromatin architecture but is associated with distinct GRF sites in different species, consistent with changes in the global GRF repertoire. This is most notable in proteasome genes, which are uniformly associated with wide/deep NFRs but are depleted of Poly(dA:dT) tracts (Figure 2E). The establishment of NFRs at these genes has likely transitioned from a mechanism dependent on the CACGAC sequence in the *Candida* clade to an Abf1-dependent mechanism in later lineages, with additional contribution from Reb1 and Rsc3/30 sites, as these GRFs gained dominance in specific species and clades (Figures 3C and S15E). Although the specific GRF mechanism underlying NFRs in proteasome genes has diverged, the establishment of wide/deep NFRs by a GRF-regulated mechanism (rather than polyA/constitutive mechanism) is conserved in all species. We hypothesize that GRF-regulated NFRs at proteasome genes may be related to the unusual transcriptional regulation of proteasome genes: these are among the few highly expressed “growth” genes (with open accessible promoters) that are further *upregulated* (rather than downregulated) during stress responses [63].

### Transition From Constitutively Programmed to GRF-Regulated NFRs in RNA Polymerase and Nuclear Pore Genes

Could promoters evolve from having constitutively programmed NFRs to regulated ones? To test this, we searched for gene sets where chromatin organization is conserved, while the underlying anti-nucleosomal sequences have diverged in a phylogenetically coherent pattern. We found that genes encoding RNA polymerase subunits exhibit deep NFRs across most of the phylogeny (Figure S15D). These genes' promoters are associated with Poly(dA:dT) tracts in *Y. lipolytica* and the species of the *Candida* clade, with both Poly(dA:dT) and the site for the Abf1 GRF in species from *S. kluyveryi* to *S. bayanus*, and only with Abf1 in the clade spanning *S. mikatae*, *S. paradoxus*, and *S. cerevisiae* (Figures 3D and S15D). Similar behavior is seen at a number of other gene sets, such as those encoding nuclear pore components (unpublished analysis). This profile suggests an evolutionary scenario where the ancestral mechanism relied on Poly(dA:dT). With the emergence of Abf1 in the LCA of the pre- and post-WGD species [34], it gained additional control of the NFRs in this gene set, alongside Poly(dA:dT) tracts. Then, after the divergence of *S. bayanus*, Poly(dA:dT) tracts were lost from the genes' promoters, leading to a complete switch from a constitutively programmed to a regulated NFRs. This compensatory evolution is consistent with patterns observed for TF binding sites in functional regulons [3],[62] and with the global transitions in GRFs described above.

### Changes in GRFs Contribute to Chromatin Divergence Between Species in Peroxisomal Genes

In some cases, the gain or loss of binding sites for GRFs can contribute to divergence in chromatin organization, coupled to phenotypic changes. Most notably, peroxisomal genes are associated with wider NFRs in *Y. lipolytica*, *C. albicans*, and *D. hansenii*, and shorter NFRs in subsequently divergent species (Figures 3E and S15F), but are not associated with intrinsic anti-nucleosomal poly(dA:dT) tracts in any of the 12 species. Instead, we find that these genes' promoters are enriched for PolyG and Rsc3/30-like sites in *Y. lipolytica*, *C. albicans*, and *D. hansenii*, but not in other species. This suggests an evolutionary scenario where either a Rsc-like motif or PolyG-based nucleosome depletion was the ancestral mechanism controlling peroxisomal genes, and was subsequently lost in the LCA of the clade spanning *S. kluyverii* and *S. cerevisiae*. This scenario is consistent with the higher expression of peroxisomal genes in *Y. lipolytica* (where peroxisomes are particularly central for carbon metabolism) and *C. albicans* (where peroxisomes play a key role in virulence).

### Evolutionary Re-Positioning of TF Motifs Relative to NFRs Contributes to Divergence of Gene Regulation in Mating, Meiosis, and Respiration Functions

Even when NFR positions and their underlying mechanisms are largely conserved, they can play an important role in regulatory divergence. Nucleosomes are generally inhibitory to TF binding [13], and in *S. cerevisiae* most functional TF binding motifs are found in NFRs [23]. Precise positioning of TF binding sites relative to nucleosomes has regulatory consequences such as changing signaling thresholds [64] or logic gating [65]. We therefore hypothesized that an evolutionary change in the location of TF-binding motifs relative to the nucleosomes in a gene's promoter can lead to regulatory divergence between species.

**Figure 4:**
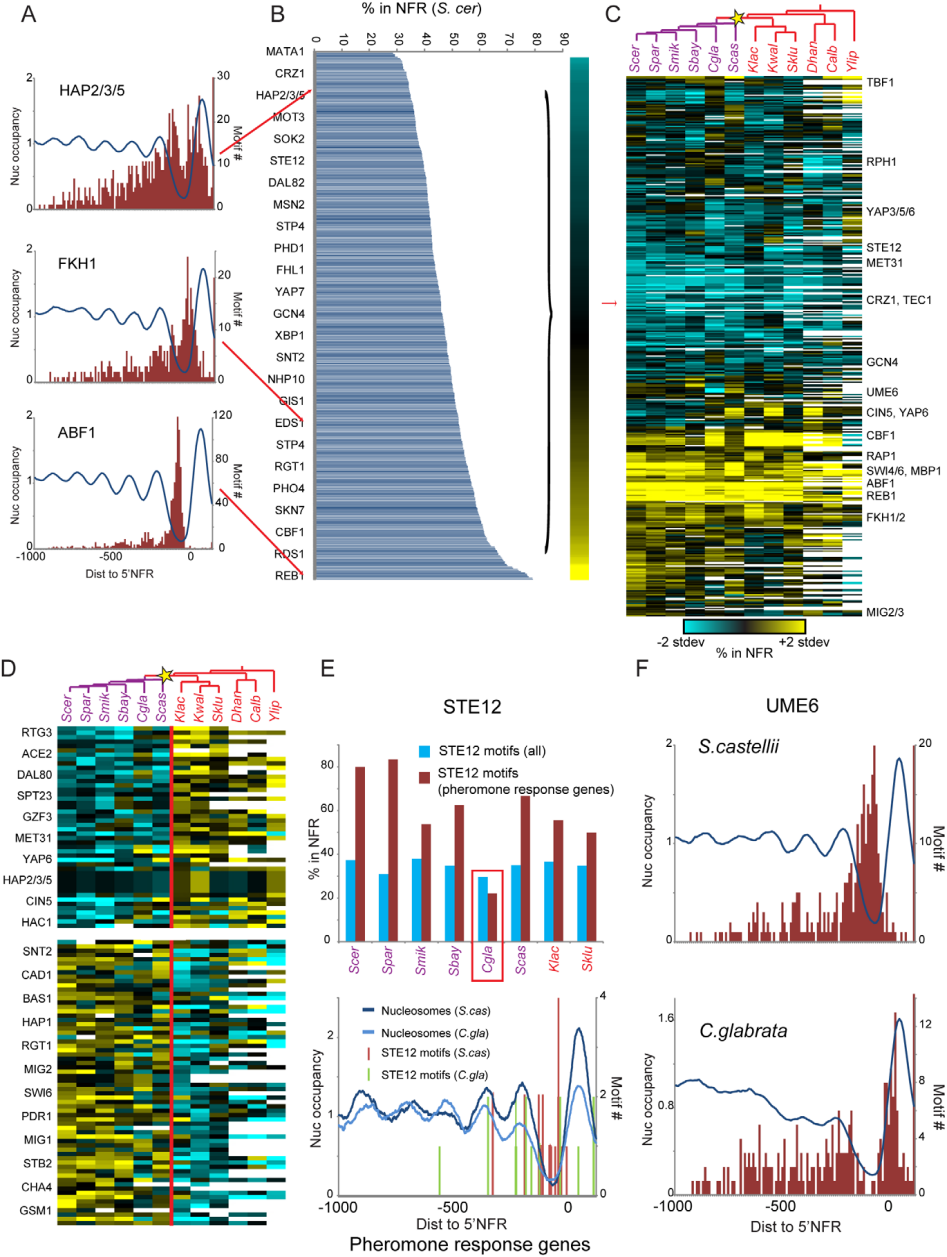
Evolutionary re-positioning of TF motif sites relative to nucleosomes. (A) Motif site location relative to chromatin features in *S. cerevisiae*. Shown are distributions of locations of the indicated TF binding sites (red), relative to the averaged chromatin profile for genes bearing instances of these sites (blue) in *S. cerevisiae*. (B) Fraction of TF binding sites located in the NFR in *S. cerevisiae* was calculated for 435 motifs, and TFs are arranged from NFR-depleted (top) to NFR-enriched (bottom). Red arrows point to TFs displayed in (A). (C) Location of TF binding sites relative to NFRs in all 12 species. Blue, NFR depleted; yellow, NFR enriched. Since the fraction of sites in NFRs varies with average NFR width and phylogenetic distance from *S. cerevisiae*, the fraction of motif instances located in NFR for each species was normalized by each species' mean and standard deviation. White, *S. cerevisiae* motifs for TFs whose orthologs are absent from a given species. (D) Motif repositioning at the WGD. Shown are the most significantly repositioned motifs between pre- and post-WGD species (*t*-test) from NFRs to nucleosomes (top) and vice versa (bottom). Star, WGD. Blue, NFR depleted; yellow, NFR enriched; values were first normalized as in panel A, and then each row was mean-normalized for visual emphasis. (E,F) Repositioning of TF binding sites relative to NFRs in *C. glabrata* meiosis and mating genes. (E) Top panel: Fraction of STE12 sites in NFRs genome-wide (blue) or at pheromone-response genes (red) for species where STE12 motif instances are enriched upstream of this gene set (*P*<10^−3^, Hypergeometric test). Bottom panel: Nucleosome data and STE12 sites location shown as in (A) for pheromone response genes in *S. castellii* and *C. glabrata*. (F) Distributions of locations of the UME6 binding site (red), relative to the averaged chromatin profile for genes bearing instances of these sites (blue) in *S. castellii* and *C. glabrata*.

To test this hypothesis, we examined the location of known TF binding motifs (from *S. cerevisiae*; [58],[59],[60],[66]) relative to nucleosome positions in each of the 12 species (Materials and Methods). Consistent with our expectations, in *S. cerevisiae* (Figure 4A,B), up to 90% of the binding sites for growth-related TFs are localized to NFRs (e.g., REB1, ABF1, RAP1, and FHL1), whereas as few as 25% of sites for stress-related TFs are at NFRs (e.g., HSF1, YAP6, HAP2/3/5, GZF3, and CRZ1). Thus, sequences that are mostly occluded by nucleosomes tend to be the binding sites for inactive TFs, and we can use chromatin information to infer TF activity under our growth conditions in each species. We therefore calculated for each motif the fraction of its instances located in NFRs in each of the 12 species (Figures 4C and S16).

The NFR positioning of many key motifs is strongly conserved. For example, sites for growth-related factors such as SWI4/6 and GCN4 were similarly NFR-exposed in all species in this phylogeny. Notably, this conservation is observed despite the fact that many motifs, which were experimentally defined for *S. cerevisiae* proteins, were globally less NFR-localized in distantly related species (Figure 4C, Figure S16B). This can be attributed in some cases to divergence of binding site preferences of the cognate TFs, and in other cases to the absence of the TF's ortholog from the genome (Figure 4C, white). Nevertheless, many motifs showed robust conserved positioning in NFRs.

Conversely, the motifs for key TFs associated with regulation of respiration and carbohydrate metabolism have repositioned relative to NFRs at the WGD, consistent with regulatory divergence in these functions (Figure 4D). For example, the sites for the HAP2/3/4/5 complex (a regulator of respiration genes) and for YAP6 (a regulator of oxidative functions) have re-positioned from NFRs to nucleosome-occluded positions post-WGD, consistent with the reduction in expression of respirative genes. In contrast, the sites for the carbon catabolite repressor MIG2 and for the glucose-responsive TF RGT1 have repositioned from nucleosomes into NFRs in post-WGD species, consistent with these factors' role in establishing a fermentative strategy through gene repression.

Motif re-positioning has also occurred at other phylogenetic points and gene sets, suggesting that this is a general regulatory and evolutionary mechanism (Figure 4E,F). For example, the mating-related STE12 motif is significantly enriched upstream of reproduction and mating-related genes in species from *S. cerevisiae* to *S. kluyverii*, including *C. glabrata*. Although STE12 sites are found in NFRs at mating genes for most of these species, they are largely nucleosome-occluded in *C. glabrata* (Figure 4E), an organism which has never been observed to mate [67]. We speculate that occlusion of STE12 sites under nucleosomes may contribute to this species' reluctance to mate, but the continued enrichment of STE12 upstream of mating genes and the retention of many meiosis-related genes [34] in *C. glabrata* suggests that it may still be capable of mating under special conditions. We therefore predict that conditions (environmental or perhaps genetic) that either mobilize or destabilize the nucleosomes covering STE12 sites at pheromone-response genes might enable mating in this species. Similarly, motifs for UME6, a major regulator of meiosis genes in *S. cerevisiae* [68], are globally NFR-positioned in all species except *C. glabrata* (Figure 4F), despite the fact that UME6 sites are enriched upstream of orthologs of meiosis-related genes in *C. glabrata*. Thus, the relative re-positioning of NFRs and TF binding sites may help explain the molecular underpinnings of dramatic changes in regulatory and phenotypic evolution.

### Duplication of TF Genes Increases the Regulatory Capacity of Conserved *Cis*-Regulatory Sites Positioned at NFRs

**Figure 5:**
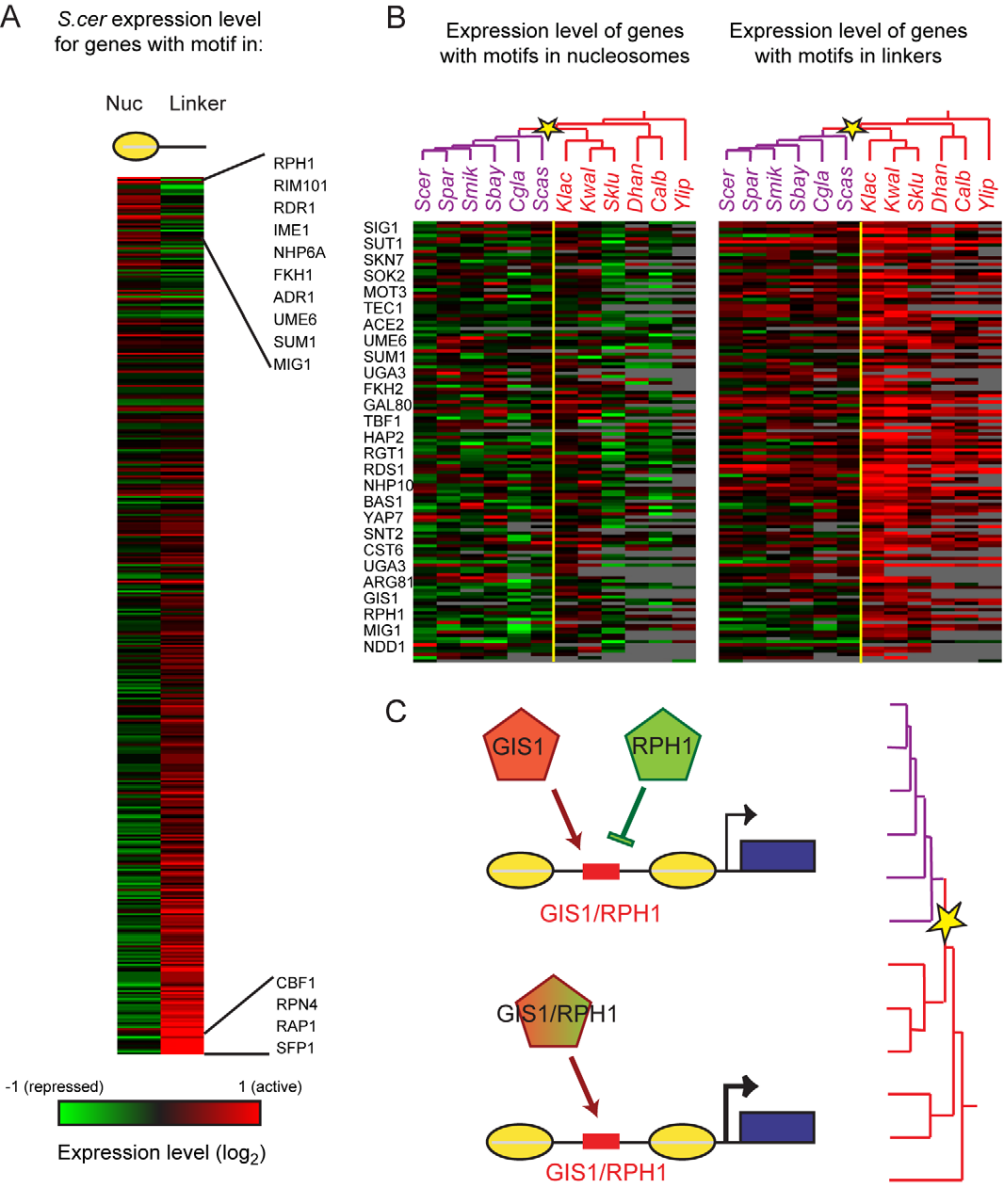
Evolution of transcription factor activity is reflected in divergence of activity of NFR-localized binding sites. (A) Nucleosome positions can be used to infer the positive or negative role of TFs in transcriptional control in *S. cerevisiae*. Average expression (mRNA abundance) of all genes with a given motif instance located in promoter nucleosomes (left) or NFRs (right). TFs are ordered by expression difference between NFR and nucleosomal binding sites, revealing transcriptional activators (bottom) and repressors (top) known to be active in these growth conditions. (B) Chromatin information reveals repressors associated with post-WGD nutrient control. For each species (columns) and each motif (rows), shown are mean expression levels of genes with the motif in nucleosomes (left matrix) or in linkers (right matrix). Shown are only the 138 motifs with increased activity in pre-WGD species [a correlation of over 0.5 to the vector (0,0,0,0,0,0,1,1,1,1,1,1)]. A small number of motifs were associated with higher activity in post-WGD species (unpublished data). Yellow star, WGD. (C) A model of increased regulatory capacity. Pre-WGD, only an single (activator-like) TF was present (GIS1/RPH1, bottom). Post-WGD (star), two paralogous TFs with the same sequence specificity are present in the genome (GIS1, RPH1, top), one is an activator (red), and the other a repressor (green).

Finally, we asked whether chromatin information could be used to infer the regulatory effect of exposed TF binding sites from the expression level of their target genes. We expect exposed TF binding sites to have different regulatory consequences depending on whether or not the TF is active and whether it acts as an activator or a repressor. We reasoned that an NFR-positioned site for an active positive regulator will be associated with a higher expression of the target genes. Conversely, an NFR-positioned site for an active negative regulator will be associated with a lower expression of the target genes. We therefore compared the expression level of all genes where a given TF motif was located within nucleosomes versus those in which the motif was located within promoter linkers (largely the NFR, Figure 5A). Consistent with our expectation, in *S. cerevisiae*, transcriptional activators known to be active in mid-log phase, such as RPN4 or PBF1, were associated with higher expression levels at genes carrying an accessible, linker-positioned motif. In contrast, NFR-positioned motifs for transcriptional repressors known to be active in mid-log (e.g., MIG1, SUM1, NRG1, DIG1, STB1/2, or RIM101; Figure 5A) were associated with lower downstream gene expression. Thus, we devised a novel approach to predict whether a given motif is associated with an activator or repressor in vivo in the growth condition tested.

When we extended this analysis to all 12 species (Figure S17), we found substantial divergence in the regulatory logic of the same NFR-positioned motif, most notably at the WGD (Figure 5B). We found a host of motifs which, when present in NFRs, were associated with differences in RNA expression levels between pre- and post-WGD species. Many of those (∼100) appeared to shift from activator-like behavior in pre-WGD species (higher target expression when in NFR) to repressor-like behavior in post-WGD species (lower target expression when in NFR). These included sites for a surprisingly large number of TFs involved in repression of metabolic genes in *S. cerevisiae*, including MIG1, GIS1, RGT1, and GAL80. Interestingly, several of these genes are found in a single copy in pre-WGD species but were retained as duplicates [34] with similar DNA-binding specificity following the WGD (e.g., GIS1/RPH1, RGT1/EDS1; Figure 5B,C). This suggests that widespread usage of competing activator/repressor pairs in *S. cerevisiae* may have been facilitated by the generation of such TF pairs at the WGD. Such duplication of *trans*-factors can serve as an alternative evolutionary mode to expand and evolve regulatory capacity [69] even when NFRs and motif positioning may be conserved.

## Discussion

In this work we used a comparative functional genomics approach to study the evolutionary interplay between chromatin organization, gene expression, and regulatory sequence elements. We aimed to achieve two goals: (1) understand the determinants of chromatin organization and function using comparative genomics and (2) characterize the role of chromatin organization in the evolution of gene regulation.

### A Comparative Approach to Study the Determinants of Chromatin Organization

What establishes the nucleosomal organization of a genome? While it has been argued that intrinsic DNA sequence can almost fully explain nucleosome organization [21], recent analysis of in vitro reconstitution data showed that the major intrinsic contributor to nucleosome positioning in budding yeast is the anti-nucleosomal behavior of Poly(dA:dT) and related sequences [21],[24],[70]. Conversely, recent reports indicate that in *S. pombe* Poly(dA:dT) plays only a minor role in nucleosome exclusion in vivo [33], indicating that even the best-understood sequence contributor to chromatin organization plays variable roles in chromatin structure in different species.

**Figure 6:**
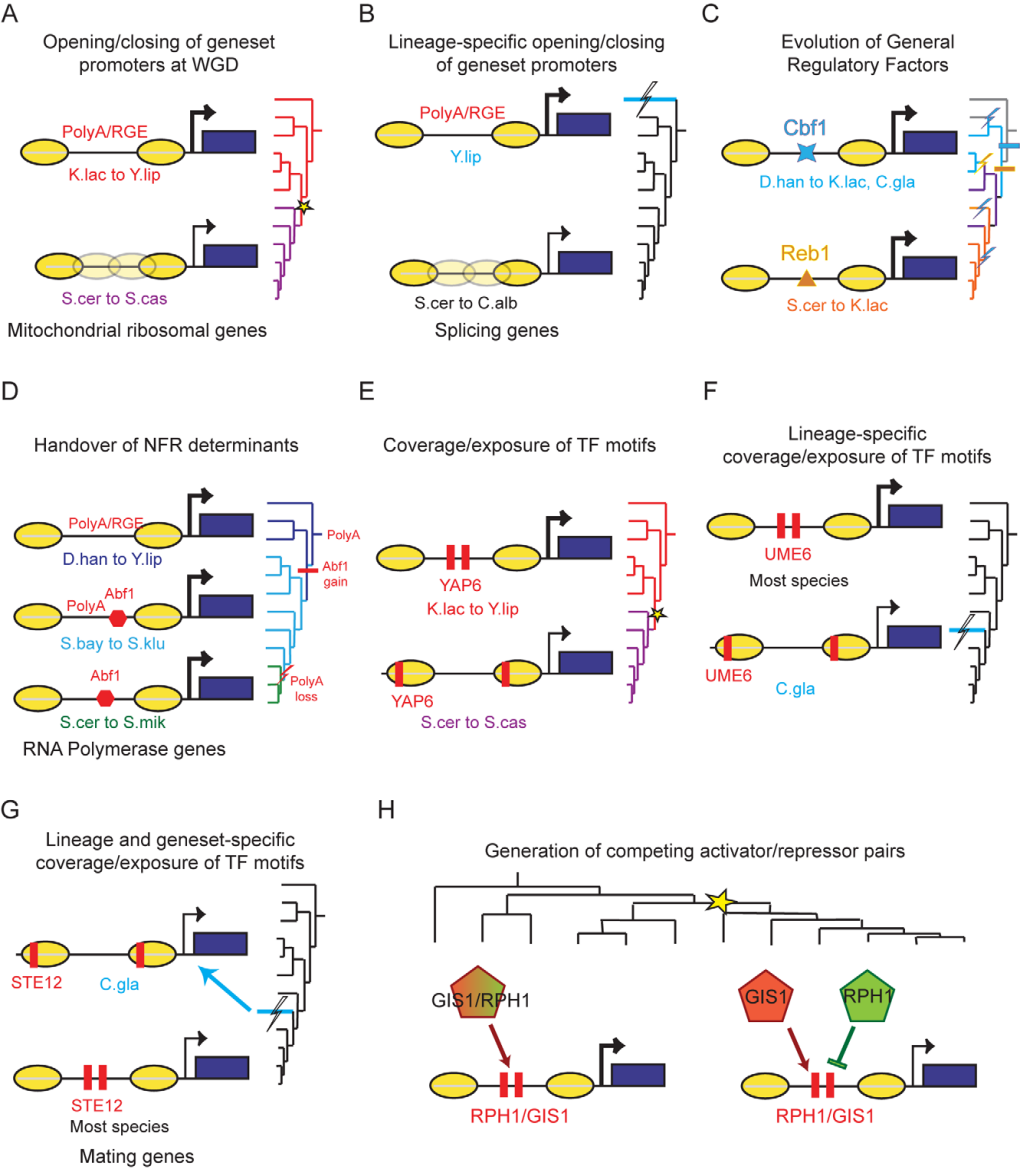
An overview of the interplay between chromatin and regulatory evolution. Shown are examples for the five key evolutionary modes discovered in the study. (A,B) Transition from “open” to “closed” NFRs associated with reduction in expression and loss of intrinsic anti-nucleosomal Poly(dA:dT) tracts in mitochondrial protein genes (at WGD) and splicing genes (after divergence of *Y. lipolytica*). (C) Global shift in usage of GRFs, resulting in a gradual transition from a Cbf1-dominated mechanism to a Reb1-dominated mechanism, through a redundant intermediate. (D) Compensatory evolution results in switch from constitutively programmed NFRs to GRF-regulated NFRs in RNA polymerase genes. (E–G) Re-positioning of motifs from NFRs to nucleosomes in oxidative functions following the WGD (E), and in meiosis and mating functions in *C. glabrata* (F,G). (H) Increased regulatory capacity at conserved NFRs and binding sites, through the duplication of *trans*-factors at the WGD.

Our analysis provides several lines of evidence that expression levels, intrinsic anti-nucleosomal sequences, and binding sites for GRFs that may recruit chromatin modifiers all play a role in establishing promoter chromatin architecture, and that the balance between these three contributors changes in evolution and between functional groups of genes. (1) We show that a sequence-based model based on in vitro depletion alone [21] can only account for 8.6%–25.7% of variance in NFR depth within any of the 12 species, including *S. cerevisiae* (10.6%). Similarly, expression levels alone can only account for 1.7%–13.1% of the variation in each species. Even when combining both the expression and intrinsic models we can only explain 13%–29% of the variation within any single species. (2) Although changes in intrinsic sequences and expression levels can explain changes in chromatin across species for some gene sets (e.g., mRPs or splicing genes; Figure 6A and B), they are insufficient to explain conserved chromatin behavior across the phylogeny (e.g., RNA Polymerase subunit genes; Figure 6D), nor do they explain changes in chromatin organization across species in other groups of genes (e.g., peroxisome genes; Figure 3E). Thus, these two determinants (alone or in combination) are insufficient to explain both intra- and inter-species variation. (3) In contrast, by comparing our in vivo data in each species to two in vitro datasets [21],[32], we find in each species a host of sequences that exhibit significantly greater nucleosome depletion in vivo than in vitro. Many of these correspond to binding sites for known GRFs that play an active role in nucleosome eviction in *S. cerevisiae* [14],[25],[56],[58], whereas others represent novel candidate GRF sequences (Figures 3 and 6C). (4) The relative contribution to nucleosome organization from GRFs, intrinsic sequences, and expression levels varies between different gene sets (in all species). For example, we show that intrinsic anti-nucleosomal sequences are enriched at NFRs in cytoplasmic RPs (in all species; Figure 2C), whereas GRFs fulfill this role in proteasome genes (in all species; Figure S15). (5) We also show that the relative contribution of one mechanism versus another can change in evolution (across species), both globally (as in the halophile *D. hansenii*, that relies more on GRFs) and in specific gene sets (as in the RNA polymerase gene set that shifted from intrinsic to regulated NFRs; Figure 6D). (6) Globally, even when we consider only the binding sites for the two best-characterized GRFs from *S. cerevisiae* (Abf1 and Reb1), GRFs alone can explain 5.2%–15.1% of the variation in nucleosome organization (in species where their orthologs are present), and 3.7%–12.6% of the residual variation after considering the contribution from expression and Poly(dA:dT). Taken together, this analysis points to a complex interplay between the different factors that control nucleosome positions, allows us to assess their contributions, and recognizes the plastic and evolvable nature of all the determinants.

### Major Modes for the Evolutionary Interplay Between Chromatin Organization and Gene Regulation

Our study also discovers an intricate and intimate relationship between conservation and divergence of chromatin organization and evolution of gene regulation. At one extreme, we found a broad functional dichotomy in chromatin organization between “growth” and “stress” genes, which is largely conserved. At the other extreme, we found that chromatin organization has diverged at a major evolutionary scale, as has happened during the evolution of respiro-fermentation, and at other points of phylogenetic and phenotypic divergence.

We found five major mechanisms by which chromatin organization can be associated with divergence of gene expression. Each of these was “used” more than once in the phylogeny, and is associated with more than one phenotypic or regulatory change, including the changes described in carbon metabolism, mating, meiosis, and splicing genes. These include (1) gain or loss of intrinsic (PolyA) sequences can open or close NFRs (Figure 6A,B) [32]; (2) conserved NFRs can be controlled by different GRF determinants, through compensatory evolution (Figure 3C); (3) NFRs can shift between constitutive and regulated determinants by compensatory (“balanced”) gain/loss of intrinsic anti-nucleosomal sequences and GRF binding sites (Figure 6D); (4) motifs can re-position relative to NFRs to change transcriptional output (Figure 6E–G); and (5) duplication and divergence of *trans*-factors can expand the regulatory behavior of conserved NFRs and binding sites (Figure 6H).

### Reprogramming Expression Through Evolution: The Case of Respiro-Fermentation

The evolution of the respiro-fermentative lifestyle following the WGD required a major reprogramming of the yeast transcriptional network and involved all of the mechanisms we describe. The shift thus included loss of intrinsic Poly(dA:dT) anti-nucleosomal sequences in some functional modules (e.g., mitochondrial RP genes), and the loss or switch of putative GRF sequences in others (e.g., oxidation-reduction genes). Furthermore, sites for certain respiratory TFs (e.g., HAP2/3/5, YAP1/3/6) have re-positioned out of NFRs, and those for glucose repression TFs have re-positioned into NFRs (e.g., RGT1, MIG1). In yet other cases, the WGD has resulted in the retention of paralogous activator-repressor pairs that control several modules in carbohydrate metabolism. Notably, each of these mechanisms has acted also at other phylogenetic points, suggesting that they point to general principles, and emphasizing the utility of the WGD as a model to study regulatory evolution.

### Prospects for Comparative Functional Genomics

Our work provides a general framework for the study of chromatin organization, function, and evolution. This includes a comprehensive genomics resource (http://www.broadinstitute.org/regev/evolfungi/) and a host of analytical approaches with broad applicability. Future studies can use our resource and methods to decipher more detailed models of the relationship between sequence elements, *trans*-factors, and gene expression, as well as on the evolution of regulatory systems. Finally, our comprehensive study in the emerging field of comparative functional genomics demonstrates how to combine the power of functional assays with extensive phylogenetic scope, to shed light both on mechanistic and evolutionary principles.

## Materials and Methods

### Strains

We used the following strains in the study: *Saccharomyces cerevisiae*, BY4741, *Saccharomyces cerevisiae*, Sigma1278b L5366, *Saccharomyces paradoxus*, NRRL Y-17217, *Saccharomyces mikatae*, IFO1815, *Saccharomyces bayanus*, NRRL Y-11845, *Candida glabrata*, CLIB 138, *Saccharomyces castellii*, NRRL Y-12630, *Kluyveromyces lactis*, CLIB 209, *Kluyveromyces waltii*, NCYC 2644, *Saccharomyces kluyveryii*, NRRL 12651, *Debaryomyces hansenii*, NCYC 2572, *Candida albicans*, SC 5314, *Yarrowia lipolytica*, CLIB 89.

### Growth Conditions

All cultures were grown in the following medium: Yeast extract (1.5%), Peptone (1%), Dextrose (2%), SC Amino Acid mix (Sunrise Science) 2 grams per liter, Adenine 100 mg/L, Tryptophan 100 mg/L, and Uracil 100 mg/L. This in-house recipe was designed to mitigate differences in growth rates between species.

### Preparation of Nucleosomal DNA and Illumina Sequencing

Overnight cultures for each species were grown in 450 ml of media at 220 RPM in a New Brunswick Scientific air-shaker at 30°C until reaching mid log-phase (OD_600_ = 0.5, WPA biowave CO 8000 Density Meter). Before formaldehyde fixation, 50 ml of the culture were transferred to a 50 ml conical and spun down immediately. The isolated cell pellets were then placed in liquid nitrogen, stored at −80°C, and were later archived in RNA later for future RNA extraction. Nucleosomal DNA isolation was carried out as previously described [23] with the following slight modifications. For different species, cells were spheroplasted with zymolase between 30 and 40 min, depending on how much time was necessary to fully remove each species' cell wall. MNase digestion levels for all samples were uniformly chosen across species to contain a slightly visible tri-nucleosome band (Figure S1). Mononucleosomes were size-selected on a gel and purified using BioRad Freeze-N-Squeeze tubes followed by phenol-chloroform extraction. Selected DNA was prepared for sequencing using the standard Illumina protocol that includes blunt ending, adaptor ligation, PCR amplification, and final size selection plus gel purification [35]. Libraries were sequenced on an Illumina 1G Analyzer, to generate 36 bp reads.

### RNA Preparation, Genomic DNA Preparation, and Labeling

Total RNA was isolated using the RNeasy Midi or Mini Kits (Qiagen) according to the provided instructions for mechanical lysis. Samples were quality controlled with the RNA 6000 Nano ll kit for the Bioanalyzer 2100 (Agilent). Genomic DNA was isolated using Genomic-tip 500/G (Qiagen) using the provided protocol for yeast. DNA samples were sheared using Covaris sonicator to 500–1000 bp fragments, as verified using DNA 7500 and DNA 12000 kit for the Bioanalyzer 2100 (Agilent). Independently sheared samples labeled with Cy3 and Cy5 were highly correlated (*R*>.97 in each of 4 independent hybridizations), indicating that the shearing procedure is reproducible and unbiased. Total RNA samples were labeled with Cy3 (cyanine fluorescent dyes) and genomic DNA samples were labeled with Cy5 using a modification of the protocol developed by Joe Derisi (UCSF) and Rosetta Inpharmatics (Kirkland, WA) that can be obtained at www.microarrays.org.

### Microarray Probe Design, Hybridization, and Normalization

Between three and four biological replicates of Cy3-labeled RNA samples were mixed with a reference Cy5 labeled genomic DNA sample and hybridized on two-color Agilent 55- or 60-mer oligo-arrays. We used the 4×44 K format for the *S. cerevisiae* strains (commercial array; 4–5 probes per target gene) or a custom 8×15 K format for all other species (2 probes per target gene, designed using eArray software, Agilent). After hybridization and washing per Agilent's instructions, arrays were scanned using an Agilent scanner and analyzed with Agilent's feature extraction software version 10.5.1.1. For each probe, the median signal intensities were background subtracted for both channels and combined by taking the log2 of the Cy3 to Cy5 ratio. To estimate the absolute expression values for each gene, we took the median of the log2 ratios across all probes. The experiments were highly reproducible; most biological replicates correlated at *R* = 0.99 and replicates with *R*<0.95 were removed. Different biological replicates were combined using quantile normalization to estimate the absolute expression level per gene per species.

### Sequencing Read Alignment and Data Post-Processing

We used BLAT [71] to map sequenced reads from each experiment to the corresponding reference genome, keeping only reads that mapped to a unique location and allowing for up to 4 mismatches. Each uniquely mapped read was then extended to a length of 100 bp. To generate a genomic nucleosome occupancy landscape, we summed all extended reads covering each base pair. We then masked all repetitive regions along each track, defining repetitive regions as locations in the genome that cannot be uniquely defined by the length of a read (36 bp). We also masked all regions of nucleosome occupancy greater than 10 times the median occupancy, to remove outlier effects that occur in places such as the rDNA locus. To normalize for sequencing depth for each genomic nucleosome track, we divided the occupancy at each location by the mean nucleosome occupancy per base pair. These normalized maps were used to generate the average nucleosome occupancy plots (Figures 1, 2A, and S2–S3).

### Detection of Nucleosome Positions

To infer the location of nucleosomes from the data, we used a Parzen window approach similar to that previously described [35],[46]. Our modified approach uses three parameters—the average DNA fragment length, the standard deviation of the Parzen window, and the maximum allowable overlap between nucleosomes. To estimate the mean DNA fragment length in each experiment, we shifted reads from one strand and then correlated them with the reads of the opposite strand. For each species, we observed a peak in the cross-correlation at a shift between 127 and 153 bp, which we used to estimate the mean DNA fragment length per experiment. We chose a standard deviation of the Parzen window of 30 bp for all species, since it closely matched the observed standard deviation around the cross-correlation peak of each experiment. Finally, we set the maximum allowable overlap between nucleosomes to 20 bp. We then shifted all read start locations by half of the mean DNA fragment length in the direction towards the dyad of the nucleosome they represent. Our approach places a normal distribution with a standard deviation of 30 bp at each read's shifted location. Summing all individual curves for all loci leads to a smoothed probability landscape of nucleosome occupancy. We next identify all peaks along the landscape, which represent nucleosome centers. The algorithm then places nucleosomes along the genome in the order of decreasing peak heights (greedy approach) and iteratively masks out these regions to prevent more than 20 bp overlap between nucleosomes.

### Finding 5′ and 3′ NFRs

We define 5′ and 3′ NFRs as the linker DNA of “significant length” closest to the 5′ and 3′ end of each gene, respectively. To find NFRs, we first created a nucleosome call landscape for each genome, normalized for sequencing depth in the same manner as the nucleosome occupancy maps (above). NFR boundaries were often obscured by very low occupancy nucleosome calls. We therefore removed all nucleosome calls with occupancy less than 40% of the average nucleosome occupancy from the map. We searched for 5′ or 3′ NFRs within 1,000 bases upstream/downstream of the 5′ or 3′ end of each gene, truncated when neighboring ORFs overlapped this region. We then defined an NFR as the linker DNA longer than 60 bp closest to the 5′ or 3′ end of each gene. If no linker longer than 60 bp was found in this search, we defined the NFR as the first linker from the 5′ or 3′ end. Our method was highly predictive of transcription start sites (TSSs) in *S. cerevisiae* [50]—the NFR boundary closest to the 5′ end of the gene was able to predict 84% of TSSs within 50 bp. Linker lengths of 50 bp or 70 bp and occupancy thresholds of 30% or 50% produced highly similar results (unpublished data).

### Controlling for Cross-Species Variation in 5′NFR-ATG Distance

Since 5′NFR-ATG distances vary substantially between species, an analysis of nucleosome organization that relies on alignment by ATG can be misleading. For example, the average nucleosome organization of *C. glabrata* and *S. castellii* look similar when aligned by the +1 nucleosomes (*Nuc^+1^*) but very different when aligned by ATG (unpublished data). A previous study [32] defines a promoter nucleosome depleted region (PNDR) score as mean nucleosome occupancy of the most depleted 100 bp region within 200 bp upstream of the ATG. Since some species have longer 5′NFR-ATG distances we reasoned that the NFR of some genes may not be contained within a 200 bp window (e.g., only a third of *C. glabrata* NFRs are contained within 200 bp, while 90% are contained within 500 bp). To avoid such pitfalls and analyze nucleosome organization consistently in all species we aligned the data by *Nuc^+1^*, which is consistent with alignment by TSS.

### Functional Gene Sets

For *S. cerevisiae* we used functional gene sets from several sources: KEGG [72], GO categories [73], MIPS [74], and BioCyc [75], as previously described [34]. For all other species, we project these gene sets based on gene orthologies [34] using the ortholog mapping at www.broad.mit.edu/regev/orthogroups.

### Chromatin Features and K-S Functional Enrichments

The chromatin features used in our analysis are listed and defined in Table S1. To quantify the enrichment for a given feature within a functional category we used the two-sample K-S test. For each K-S test, we defined our two sample sets as genes within a given functional group and all other genes in the genome. The K-S test quantifies the distance between the distributions of a given chromatin feature for the two sets. The K-S statistic *K_K-S_* is defined as the maximum absolute difference between the cumulative distribution functions (CDFs) of the two samples. We estimated the *P* value, *P_K-S_*, for the statistical significance of this difference as follows:
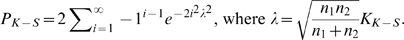



For further analysis, we converted *P* values to K-S scores, *S_K-S_*, where *S_K-S_* = ±log_10_(*P_K-S_*) if the difference realizing the statistic *K_K-S_* is positive/negative, respectively. To account for multiple hypotheses testing, we only considered *P_K-S_* as significant if it was below the *P* value threshold for a False Discovery Rate of 5% [76]. This analysis was also applied to absolute expression levels, Poly(dA:dT) strength in NFRs, *trans* factor motif affinity scores in NFRs, and comparison in expression of sites located in NFRs versus sites located in nucleosomes.

### Lowess Smoothing

To subtract the effect of expression on observed chromatin features, we used robust Lowess smoothing. We smoothed the scatter data of each chromatin feature versus expression level using a Lowess linear fit and a smoothing window set to 10% of the span of expression level values. We assigned zero weight to outliers, defined as data more than six standard deviations from the mean. To remove the effect of expression, the Lowess fit was subtracted from its corresponding chromatin feature value. K-S functional enrichments for the Lowess subtracted chromatin features were calculated as described above.

### Quantification of Contribution of Three Determinants on NFR Occupancy

We assessed transcriptional activity by absolute RNA expression. We assessed intrinsic anti-nucleosomal sequence by Poly(dA:dT) strength in NFRs, since it explains the vast majority of the intrinsic sequence information and generalizes to all species in an unbiased manner. Other models of intrinsic sequence contribution [21],[48] yielded similar results (unpublished data). We assess the contribution of chromatin modifiers based on the Abf1 and Reb1 motif affinity scores in NFRs. This is a conservative estimate, since we only considered the two most established GRFs. To quantify the contribution of each of these factors on NFR occupancy, we used robust Lowess smoothing as described above.

To compute the percent of variance explained by the robust Lowess fit, each NFR occupancy was assigned a “fitted” value F_i_ from the Lowess fitting line based on each of the three determinants. Then the variance of the residuals σ^2^_R_ = var({F_i_−Z_i_}) was compared to the variance of the original data σ^2^_D_ = var({Z_i_}). The percent of variance explained is defined as (1−(σ^2^_R_/σ^2^_D_))*100. To find the percent variance explained by all determinants we first fit NFR occupancy versus one determinant, then iteratively took the residual, and fit it against the next determinant. For the figures, we first fit expression, then fit the successive residual versus Poly(dA:dT) tracts, and then fit the residual versus Abf1 and Reb1 motif affinity scores. Changing the order of the successive fits did not significantly reduce the total percent variance explained.

### Promoter TF Motif Scanning

Promoter sequences for each gene were defined as 1,000 bases upstream, truncated when neighboring ORFs overlapped with this region. We collected a library of Position Weight Matrices (PWMs) for several hundred *S. cerevisiae* DNA-binding proteins as previously defined [58],[59],[60],[66]. Motif targets were identified via the TestMOTIF software program [77] using a 3-order Markov background model estimated from the entire set of promoters per genome. We considered all motif instances with *P* value <0.05 as significant. Since a few motifs had thousands of instances for this cutoff, we also limited the number of promoters with significant sites to the top 1,000. The upper bound was chosen to exceed the maximal number of promoters bound (866, *P* value <0.05) by any TF in *S. cerevisiae*, as measured by ChIP-chip [60]. For all subsequent motif analyses, we used the above criterion to define two sets of sites: (1) all significant sites within allowed promoters and (2) the best sites per allowed promoters.

### Global Motif Analysis

All motif instances were binned into five regions (*Nuc^+1^*, 5′NFR, *Nuc^−1^*, *Nuc^−2^*_,_ and NFR2—the linker between *Nuc* ^−*1*^ and *Nuc* ^−*2*^) if their centers overlapped with the defined regions. In addition, sites were also split into two categories: *Linkers* (5′NFR and NFR2) and *Nucs* (*Nuc^+1^*, *Nuc* ^−*1*^, and *Nuc* ^−*2*^). We assigned the expression level of each gene to each site in the upstream promoter of that gene. We used a two-sample K-S test (as described above) to quantify the difference in expression levels between sites in *Linkers* versus *Nucs*.

To quantify the preference of a motif for nucleosome depleted regions, we compared the mean log2 normalized nucleosome occupancy at all sites (x) against the mean log2 normalized nucleosome occupancy over the corresponding promoters (y). To estimate the significance of the difference of the two vectors (x-y), we used the paired Wilcoxon signed rank test that assigns a *P* value for rejecting the null hypothesis that x-y comes from a continuous, symmetric distribution with a zero median.

### Motif GO Enrichments

To estimate the probability that *k* or more elements intersect subsets of *n* and *m* members at random in a superset of size *N* (or the *P* value for overlap of *k*, *P_HG_*) we summed over the right tail of a hypergeometric distribution:
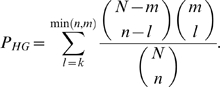



Using the hypergeometric *P* values, we estimated the significance of *k* overlaps between *n* genes with sites in their upstream promoter and *m* genes within a GO category, for a species with *N* genes.

### Scoring Motif Occurrences in NFRs

We represent each motif of length *L* by a position specific scoring matrix (PSSM) *P*, or the probability distribution *P(S_1_, …, S_L_)* of that motif occurring over any sequence *S_1_…S_L_*. This is a standard approximation to a factor binding energy for sequence *S_1_…S_L_*. We also learned the 0^th^-order Markov background probability distribution *B(S_1_, …, S_L_)* for each sequence *S_1_*…*S_L_*, set to the frequency of the four nucleotides in the promoter regions of a given species. We calculate *A(P,S)*, a motif's affinity score for an NFR sequence *S*, by summing the contributions of *P(S_1_, …, S_L_)/B(S_1_, …, S_L_)* over all allowable positions *k* in *S* as follows:
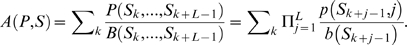



Here, *b(S_k+j-1_)* is the background probability of the nucleotide *S_k+j-1_* of sequence *S*, and *p(S_k+j-1_,j)* is the probability for nucleotide *S_k+j-1_* in position *j* of the motif's PSSM. For the results in this study, we combined the contributions of both forward and reverse strands of each NFR. Also, normalizing the affinity by the length of each NFR sequence did not affect our results significantly.

### N-mer Analysis

Prior to analysis, we log2-transformed the normalized nucleosome occupancy data (Data post-processing, above), subtracted the mean, and divided by the standard deviation. Hence, the global nucleosome occupancy data for each species is approximately normal with zero mean and unit variance. We also used the same procedure for processing published in vitro data [21].

For each N-mer, we define the *in vivo depletion score* as the mean −log2 normalized nucleosome occupancy across all instances and all instances of the reverse complement. We also defined the *depletion score relative to in vitro* as power 2 of the difference between the in vivo depletion scores in each species and the in vitro depletion scores in *S. cerevisiae* (also repeated for in vitro data from *C. albicans* [32]). The analysis was done for *N* = 5, 6, 7, 8 and also repeated for N-mers found only in coding regions and only in upstream promoter regions.

### Poly(dA:dT) Tracts

To annotate all Poly(dA:dT) tracts in each species and determine their nucleosome repelling strength we used an approach similar to a previously described one [48]. In summary, for each species' genome we found all PolyA or PolyT tracts of length *L* of 5 bp or more. We define the depletion score for a tract of length *L* as the mean of the −log2 normalized nucleosome occupancy across all instances of that length. This was calculated both using in vitro data from *S. cerevisiae* [21] and the in vivo data from each species. For long Poly(dA:dT) tracts with very few occurrences in a given genome we noticed a larger variation in the depletion score, likely due to small sample size. To mitigate this problem, we fit a line for depletion scores versus *L* using a weighted linear least squares fit with weights proportional to the number of occurrences for tracts of length *L*. We then used the line as an estimate for long tracts with fewer than 100 occurrences in a given genome. We iterated this procedure for all maximal Poly(dA:dT) tract with *k* allowed mismatches, *k* = 1, …,20. The depletion score increases linearly with *L* for tracts with different *k*, confirming that a linear fit is appropriate (Figure S11).

To aggregate all non-overlapping Poly(dA:dT) tracts within a given genome, we first quantized the strengths for each *L*. We define the fold depletion score of all tracts of length *L* as power 2 of the depletion score. We then quantized all Poly(dA:dT) tract fold depletion scores to the highest fold depletion level exceeding 2, 4, 8, 16, and 32. For example, a tract with a depletion score of 3.5 is 2^3.5^ = 11.3-fold depleted in nucleosomes relative to average, and would be assigned a fold depletion score of 8. We next iterated over all Poly(dA:dT) tracts with mismatches *k* = 0, …,20, replacing overlapping tracts only if the tract with more mismatches had a higher quantized fold depletion score.

### Data Availability

Data have been submitted to GEO, accession #GSE21960.

## Supporting Information

Supplementary website http://www.broadinstitute.org/regev/evolfungi/

Figure S1 **Isolation of mononucleosomal DNA from 12 species.** Shown are MNase titrations from which mononucleosomal DNA (red box) was isolated for construction of deep sequencing libraries.
(2.22 MB PDF)

Figure S2 **5′ alignment of nucleosome data for 12 species.** Sequencing reads were extended to a length of 100 bp. Data for all annotated genes were extracted and aligned by *Nuc^+1^*, and average profiles over all genes are shown for each species.
(0.60 MB PDF)

Figure S3 **3′ alignment of nucleosome data.** Data shown as in Figure S2, but aligned by *Nuc^+N^*.
(0.61 MB PDF)

Figure S4 **Non-redundant set of chromatin features.** After calling nucleosomes from *S. cerevisiae* data, 56 chromatin features were estimated at all gene promoters. Shown is the correlation matrix between all features in *S. cerevisiae*. The features used in this study are highlighted in red.
(0.52 MB PDF)

Figure S5 **Two scenarios for changes in NFR-ATG distance.** (A) Canonical promoter architecture in *S. cerevisiae* –transcriptional start site (TSS) is typically found at ∼13 nt 3′ to the upstream border of *Nuc^+1^*. (B) 5′NFR to ATG distance (*D_5′NFR-ATG_*) varies in other species without annotated TSSs. For example, NFR-ATG distance is shorter in *D. hansenii* than in *S. cerevisiae* (Figure 1E). Depending on the location of the TSS, this result is consistent with two possibilities (or any admixture thereof): (C) TSSs are located 13 nt into *Nuc^+1^*, and 5′ UTRs are globally shorter, or (D) 5′ UTRs are the same length and the TSS is situated within the NFR. Several lines of evidence support the latter possibility (D), including the conservation of 5′UTR length distribution in a small number of measured cases in *S. cerevisiae* and *C. albicans* [45], the known variation in TATA-TSS distances between *S. pombe* and *S. cerevisiae* [78], and the known variation between yeast, fly, and humans in TSS location relative to *Nuc^+1^* [26],[46],[79],[80]. Thus, it is likely that TSS location relative to *Nuc^+1^* varies substantially between *Hemiascomycota* species. This would affect TSS-exposure rates and pre-initiation complex geometry and has unknown consequences for basic gene regulatory mechanisms [16],[81].
(0.27 MB PDF)

Figure S6 **Conservation and variation in chromatin structure of functional gene sets.** (A) Global overview of chromatin behavior within functional gene sets. K-S scores (Materials and Methods) were calculated for 8 parameters for 4,774 gene sets in each species as in Figure 2A,B. Only gene sets with over 10 members in 10 or more of species are shown (1,159 gene sets, including “transcriptional modules” and genes annotated based on expression changes in deletion strains [34], both excluded from Figure 2). Gene sets were clustered by K-S scores, and enrichments are shown as in Figure 2C–J. Selected clusters of gene sets are marked on the right. Note that stress-related gene sets tend to become less enriched for various chromatin and expression features at increasing phylogenetic distance from *S. cerevisiae*, likely due to the rapid gain/loss of these genes over this phylogenetic distance [34]. Importantly, genes in distant species associated with orthogroups lacking an *S. cerevisiae* member tend to be poorly expressed and exhibit stress-related chromatin characteristics (unpublished data), indicating that these genes likely play species-specific stress-related roles. (B) Gene sets associated with increase in NFR occupancy in post-WGD species were identified and are shown as in panel A.
(0.61 MB PNG)

Figure S7 **3′ NFR enrichments.** K-S enrichments for 3′ chromatin parameters were calculated for all gene sets as in Figure 2, considering only genes in convergent (tail to tail) orientation. K-S scores were clustered as in Figure S6. Few enrichments are apparent, most notably enrichments of long 3′ NFRs (and either low *Nuc^+N^* occupancy or tight 3′ CDS spacing) downstream of ribosomal protein genes. Additional annotations associated with long 3′ NFRs are noted on the right.
(6.80 MB PDF)

Figure S8 **Chromatin feature enrichment for gene sets is robust to gene orientation.** K-S enrichments for chromatin parameters were calculated for all gene sets as in Figure 2A,B and Figure S6, but only considering genes in tandem (head to tail) orientation. Annotations are ordered as in Figure S6. The increased number of grey boxes indicates gene sets with less than 10 members when divergently oriented promoters are excluded.
(0.60 MB PNG)

Figure S9 **Relationship between RNA level, antinucleosomal tracts, GRF sites, and chromatin structure.** (A–D) Gene-by-gene comparisons of NFR depth to mRNA levels or Poly(dA:dT) signal. Shown are plots of NFR depth (*y*-axis) versus mRNA level (A,C) or Poly(dA:dT) score at the NFR (B,D) for each gene (blue dot) in the *S. cerevisiae* (A,B) or *C. albicans* (C,D) genome. Also shown is a 50-gene running window average for each panel (red). (E) Variation in NFR depth explained by each determinant and their combination. Shown are the % variation in NFR depth (bars, *y*-axis) explained in each species by each determinant alone (dark blue, polyA; red, mRNA expression; green, binding sites for Abf1 and Reb1 in the NFR) and two combinations (purple, polyA and mRNA expression; light blue, polyA, mRNA, and the GRF sites). (F) The residual contribution of polyA and GRF sites. Shown are the % variation in NFR depth (bars, *y*-axis) explained in each species by mRNA expression alone (blue bars), the subsequent residual variation explained by polyA (red bars) and the residual variation (after mRNA and polyA) explained by the binding sites for the GRFs Abf1 and Reb1 (green).
(2.97 MB PDF)

Figure S10 **Relationship between RNA level and chromatin structure.** The extent of variation in a given chromatin parameter which is explained by RNA abundance was calculated (LOWESS, Materials and Methods) for each feature in each species. The fitted LOWESS curve was then used to correct for the effect of transcription on chromatin packaging, and K-S enrichments were recalculated as in Figure 2A,B. Shown are K-S enrichments, as in Figure S6, for gene sets calculated before (“Raw”) and after (“Corrected”) LOWESS-correction. NFR occupancy enrichments are not strongly influenced by RNA levels, whereas *Nuc^+1^* occupancy and CDS nucleosome spacing enrichments were more substantially explained by RNA abundance measures.
(5.52 MB PDF)

Figure S11 **Relationship between Poly(dA:dT) tracts and nucleosome depletion varies between species.** (A,B) Shown are plots of nucleosome depletion (log_2,_ *y*-axis) versus length of Poly(dA:dT) tract (*x*-axis) for Poly(dA:dT) tracts with no mismatches (A) or 2 mismatches (B). (C) Species differ in the number of antinucleosomal PolyA tracts. Shown are the number of anti-nucleosomal Poly(dA:dT) tracts with a strength score greater than 4 in the NFRs of each species. (D) Median NFR width (per species) is correlated (*r* = 0.77) with number of anti-nucleosomal Poly(dA:dT) tracts in the NFR. Shown are the number of anti-nucleosomal Poly(dA:dT) tracts with a strength score greater than 2 in the NFRs in each species versus that species' average NFR length.
(0.37 MB PDF)

Figure S12 **The calculated effect Poly(dA:dT) tracts on nucleosome depletion for gene sets is independent of the dataset used.** We calculated the extent of nucleosome depletion over various lengths of Poly(dA:dT) using either in vitro nucleosome reconstitution data [21] (A) or our in vivo nucleosome mapping data from all studied species (B). Shown are K-S enrichments for gene sets ordered as in Figure S6.
(4.39 MB PDF)

Figure S13 **Analysis of anti-nucleosomal 6-mers.** (A) The extent of nucleosome depletion over all 6-mers was calculated as in Figure 3A. Shown is a scatter plot of the nucleosome depletion observed in in vitro reconstitutions [21] versus the maximal nucleosome depletion observed in vivo in any of the 12 species in this study. (B) In vivo nucleosome depletion of each 6-mer in *S. cerevisiae* is plotted against that in *S. mikatae*. Few differences are observed. (C) As in (B), but for *S. cerevisiae* versus *K. lactis*. CGCG-containing Rsc3/30-like motifs (green) are more nucleosome-depleted in *S. cerevisiae* than in *K. lactis*, whereas the Cbf1 motif CACGTG and related motifs (blue) are more nucleosome-depleted in *K. lactis* than in *S. cerevisiae*. This is consistent with the loss of the Rsc3/30 ortholog in *K. lactis* [82]. (D) Nucleosome depletion score for four major anti-nucleosomal 6-mers across 13 in vivo datasets and 2 in vitro datasets [21],[32].
(0.72 MB PDF)

Figure S14 **Species-specific usage of GRF-related motifs.** Shown are nucleosome depletion scores over the indicated sequences for all in vivo data reported here, and for two published in vitro reconstitution datasets (blue) in *S. cerevisiae* [21] and *C. albicans* [32]. All shown elements are associated with nucleosome depletion in at least one species.
(0.32 MB PDF)

Figure S15 **Evolution of anti-nucleosomal programming at specific gene sets.** Enrichment of Poly(dA:dT) tracts (A8) or motifs for various GRFs was calculated for the indicated gene sets. Enrichments are shown for high (red) or low (green) expression levels, high (yellow) or low (blue) 5′NFR occupancy or length, and enrichment (pink) or depletion (blue) of A8 or GRF motifs for each gene set. K-S *P* value saturation levels are indicated to the right of each panel.
(0.46 MB PDF)

Figure S16 **Re-positioning of TF motifs relative to NFRs.** (A) Location of TF binding sites relative to NFRs in all 12 species (identical to Figure 4C): blue, NFR depleted; yellow, NFR enriched. Since the fraction of sites in NFRs varies with average NFR width and phylogenetic distance from *S. cerevisiae*, the fraction of motif instances located in NFR for each species was normalized by each species' mean and standard deviation. White, *S. cerevisiae* protein motifs whose orthologs are absent from a given species. (B) As in (A) and Figure 4C, but without scaling each species. The overall fraction of motifs located in NFRs decreases with increasing phylogenetic distance from *S. cerevisiae*, likely due to variation in TF binding site affinities. Much of the variation in the overall fraction of motifs in NFRs can also be ascribed to variation in NFR length across species—*C. glabrata*, for example, exhibits unusually long NFRs (Table S3), and the resulting high motif localization to NFRs is therefore corrected by normalization to overall % NFR in panel A and Figure 4C.
(1.69 MB PDF)

Figure S17 **Divergence of activity of NFR-localized binding sites.** Variation in TF activity across the 12 species. Data shown as in Figure 5A–B, but for all TF motifs, for all species studied (columns).
(1.26 MB PDF)

Table S1 **Chromatin features.** Definitions of chromatin parameters used throughout the text.
(0.03 MB XLS)

Table S2 **Gene set enrichments for mRNA abundance.** For each species (columns) in this study, we display the K-S score (Materials and Methods) for mRNA abundance enrichment within functional gene sets (rows). GO, KEGG, MIPS, BioCyc categories with 10 or more species with valid enrichments are displayed.
(0.26 MB XLS)

Table S3 **Phylogenetic variation in global chromatin features.** For each species, we computed the median of the global distribution of each of the 56 estimated chromatin features. Features are displayed as rows, species as columns.
(0.04 MB XLS)

Table S4 **Gene set enrichments for 5′ chromatin features.** For each species (columns) in this study, we display the K-S score (Materials and Methods) for enrichment within functional gene sets (rows) of the 13 5′ chromatin features used in this study. The features are stacked from top to bottom in the following order: 5′NFR-ATG distance or *D_5_*_′*NFR-ATG*_ (5′tss_pred), 5′NFR length (5′nfrLen), 5′NFR occupancy (5′nfrOcup), Nuc^+1^ to 5′NFR occupancy (plus1_to_5′nfrOcup), Nuc^+1^ occupancy (plus1Ocup), Fuzziness of Nuc^+1^ (plus1Fuzzy), width of Nuc^+1^ (plus1Width), median adjacent spacing for Nuc^+1^ to Nuc^+4^ (plus1SpacingDown3), Nuc^−1^ to 5′NFR occupancy (minus1_to_5′nfrOcup), Nuc^−1^ occupancy (minus1Ocup), Fuzziness of Nuc^−1^ (minus1Fuzzy), width of Nuc^−1^ (minus1Width), and median adjacent spacing for Nuc^−1^ to Nuc^−4^ (minus1SpacingUp3). GO, KEGG, MIPS, BioCyc categories with 10 or more species with valid enrichments are displayed.
(2.44 MB XLS)

Table S5 **Gene set enrichments for 3′ chromatin features.** For each species (columns) in this study, we display the K-S score (Materials and Methods) for enrichment within functional gene sets (rows) of the 13 3′ chromatin features used in this study. The 13 features are stacked from top to bottom in the following order: 3′NFR-stop codon distance (3′tss_pred), 3′NFR length (3′nfrLen), 3′NFR occupancy (3′nfrOcup), Nuc^+N^ to 3′NFR occupancy (plusN_to_3^′^nfrOcup), Nuc^+N^ occupancy (plusNOcup), fuzziness of Nuc^+N^ (plusNFuzzy), width of Nuc^+N^ (plusNWidth), median adjacent spacing for Nuc^+N−3^ to Nuc^+N^ plusNSpacingDown3, Nuc^+N+1^ to 3′NFR occupancy (plusN+1_to_3′nfrOcup), Nuc^+N+1^ occupancy (plusN+1Ocup), fuzziness of Nuc^+N+1^ (plusN+1Fuzzy), width of Nuc^+N+1^ (plusN+1Width), and median adjacent spacing for Nuc^+N+1^ to Nuc^+N+4^ plusN+1SpacingUp3. GO, KEGG, MIPS, BioCyc categories with 10 or more species with valid enrichments are displayed.
(0.92 MB XLS)

Table S6 **Nucleosome depletion over k-mers.** Shown are the negative depletion scores (Materials and Methods) of all possible genomic 7-mers (rows) for each species (columns) in this study.
(4.76 MB XLS)

Table S7 **Distribution of Poly(dA:dT) tract lengths in all species.** Shown are the number of genomic occurrences of Poly(dA:dT) tracks of length *L* (rows) for all species (columns) in this study.
(0.04 MB XLS)

Table S8 **Anti-nucleosomal sequence enrichments versus gene sets.** For each species (columns) in this study, we display the K-S score (Materials and Methods) enrichment in the NFRs of functional gene sets (rows) for different intrinsic and *trans* anti-nucleosomal sequences. The anti-nucleosomal elements are stacked from top to bottom in the following order: Poly(dA:dT) in vitro strength, Poly(dA:dT) in vivo strength, PolyA8, RGE, ABF1, CBF1, REB1, RSC30, PolyG8, and *C. albicans*-specific k-mer CACGAC. GO, KEGG, MIPS, BioCyc categories with 10 or more species with valid enrichments are displayed.
(1.95 MB XLS)

## Abbreviations

BYA: billion years ago
cRPs: cytoplasmic ribosomal proteins
GRFs: general regulatory factors
K-S: Kolmogorov-Smirnov
LCA: last common ancestor
mRP: mitochondrial ribosomal protein
MYA: million years ago
NFRs: nucleosome free regions
PSSM: position specific scoring matrix
TF: transcription factor
TSSs: transcription start sites
UTR: untranslated region
