# Supplementary figures and images for: The Role of Nucleosome Positioning in the Evolution of Gene Regulation
Source: PLoS Biol. 2010 Jul 6;8(7):e1000414. doi: 10.1371/journal.pbio.1000414 (PMC2897762; doi:10.1371/journal.pbio.1000414)

Figure S1

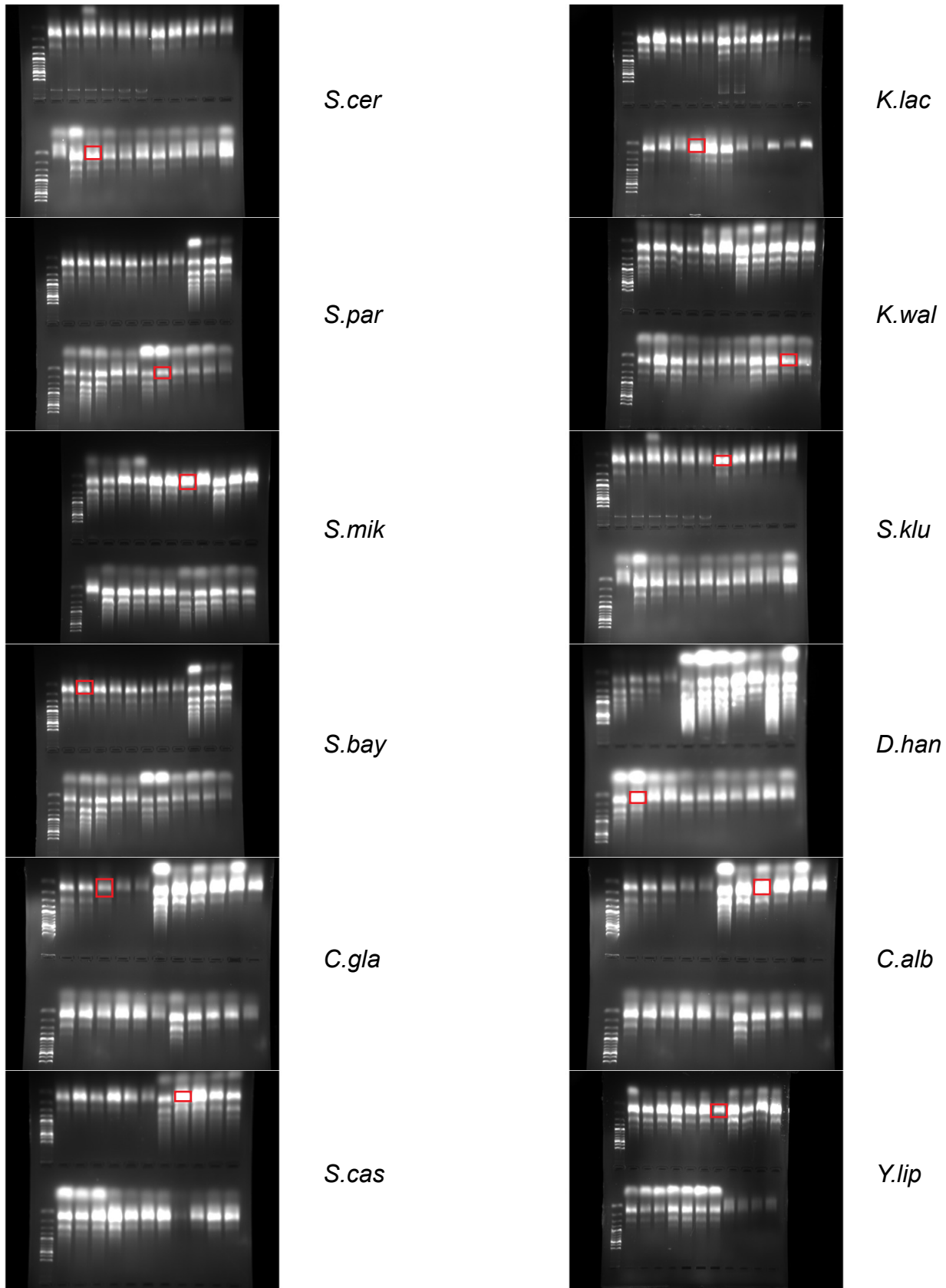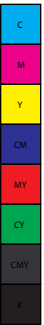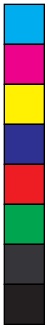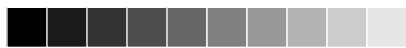

Supplement: Figure S1 — Isolation of mononucleosomal DNA from 12 species. Shown are MNase titrations from which mononucleosomal DNA (red box) was isolated for construction of deep sequencing libraries. (2.22 MB PDF) [file pbio.1000414.s001.pdf]

Figure S2

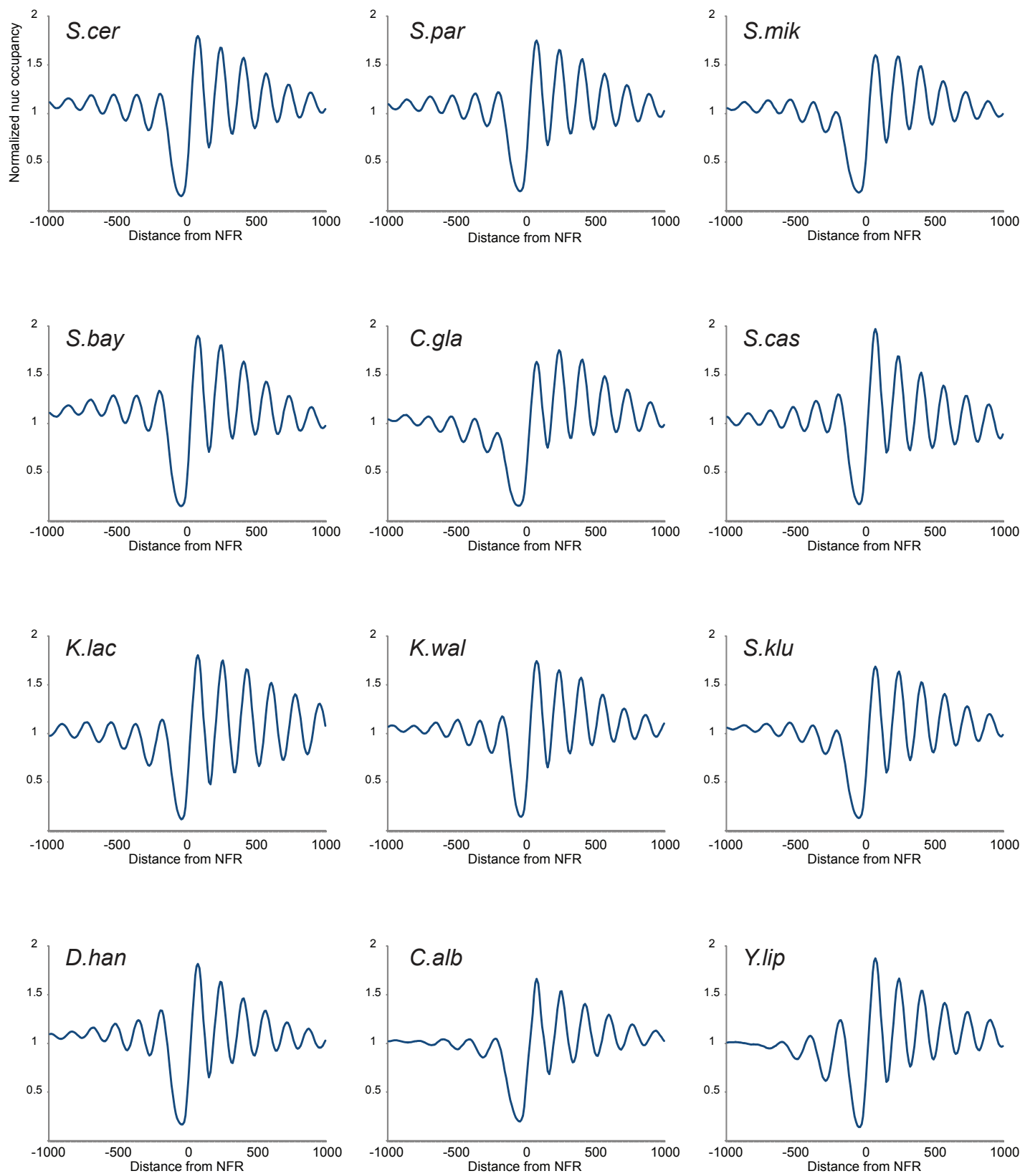

Supplement: Figure S2 — 5′ alignment of nucleosome data for 12 species. Sequencing reads were extended to a length of 100 bp. Data for all annotated genes were extracted and aligned by Nuc+1, and average profiles over all genes are shown for each species. (0.60 MB PDF) [file pbio.1000414.s002.pdf]

Figure S3

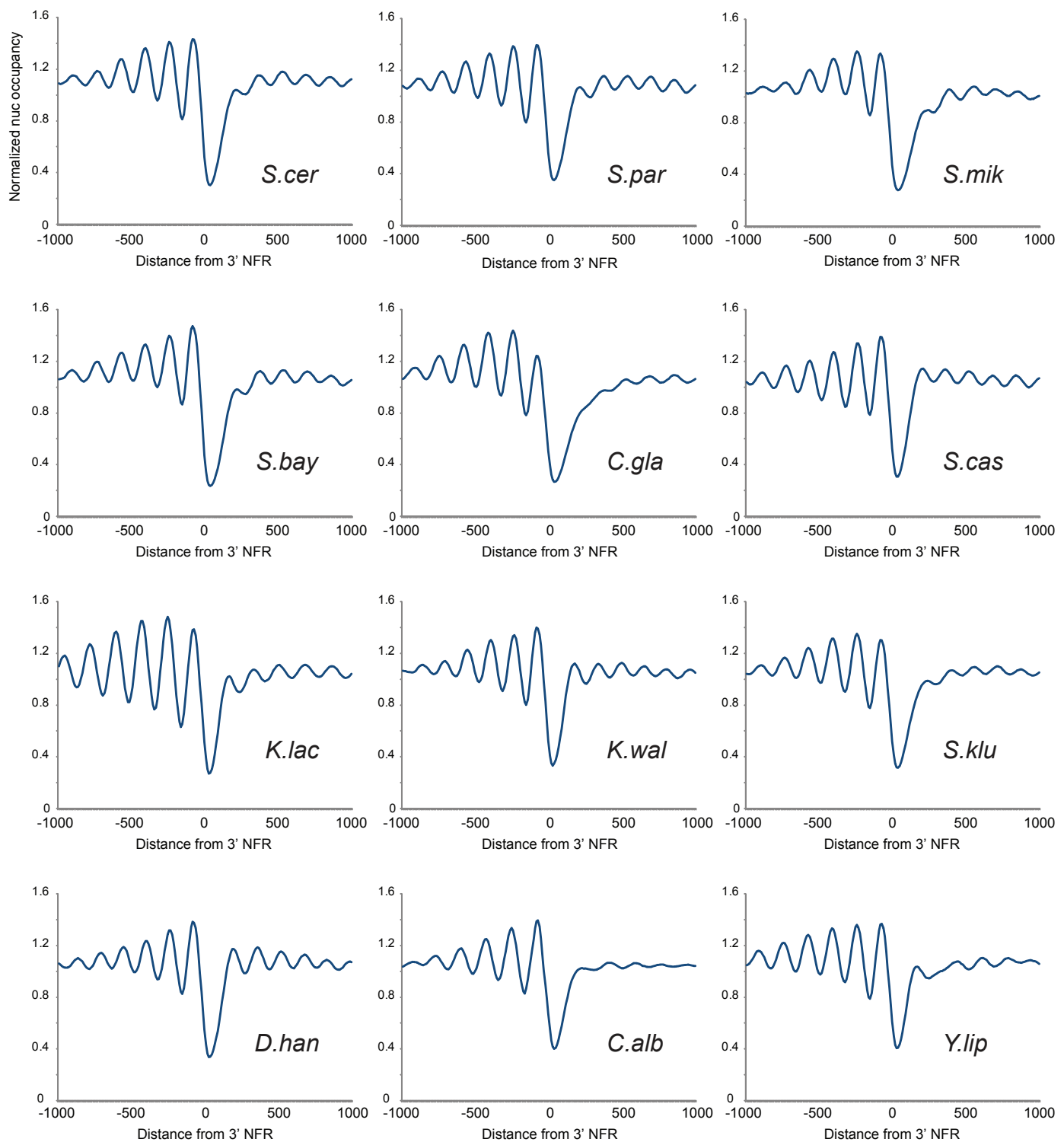

Supplement: Figure S3 — 3′ alignment of nucleosome data. Data shown as in Figure S2, but aligned by Nuc+N. (0.61 MB PDF) [file pbio.1000414.s003.pdf]

Figure S4

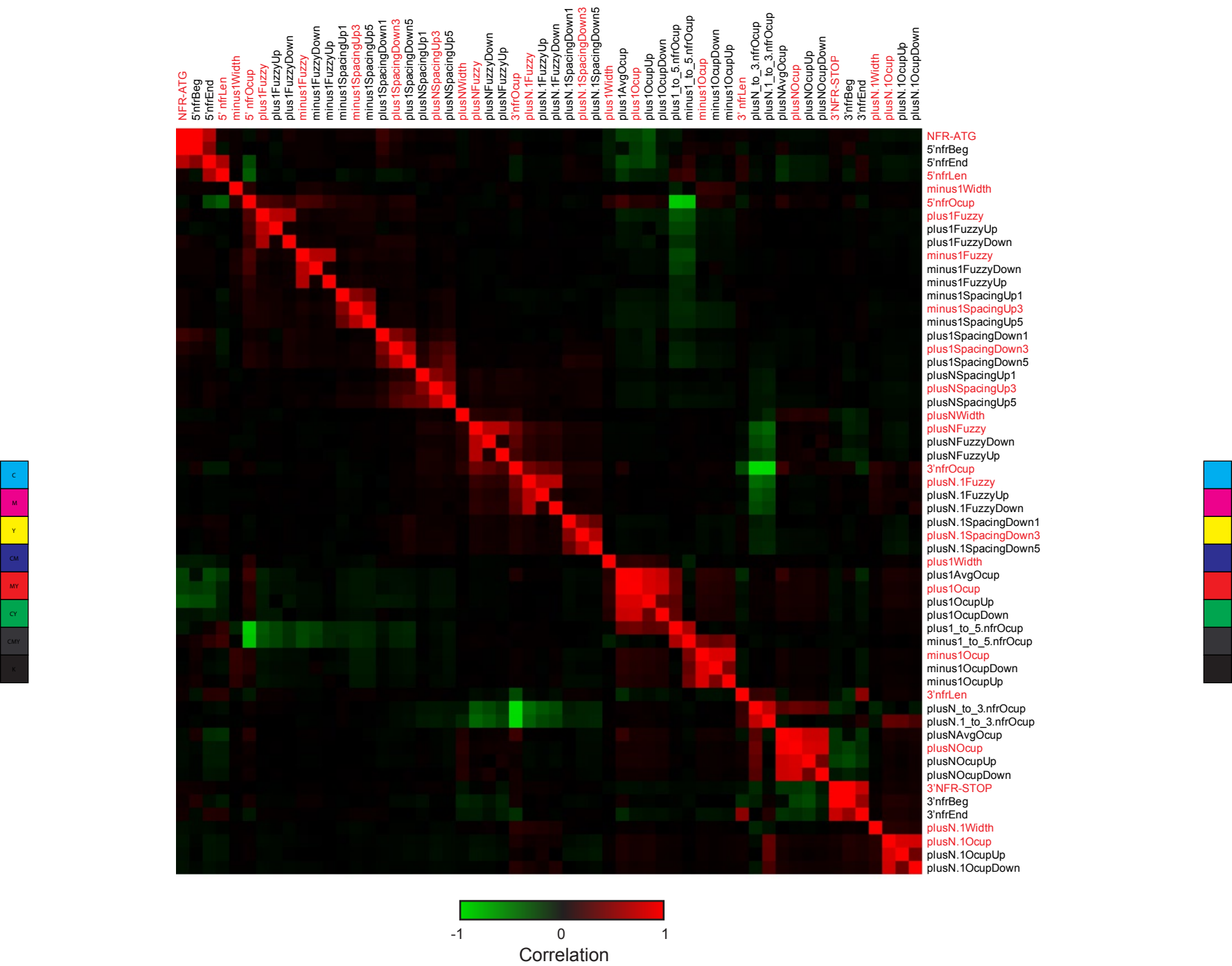

Supplement: Figure S4 — Non-redundant set of chromatin features. After calling nucleosomes from S. cerevisiae data, 56 chromatin features were estimated at all gene promoters. Shown is the correlation matrix between all features in S. cerevisiae. The features used in this study are highlighted in red. (0.52 MB PDF) [file pbio.1000414.s004.pdf]

Figure S5

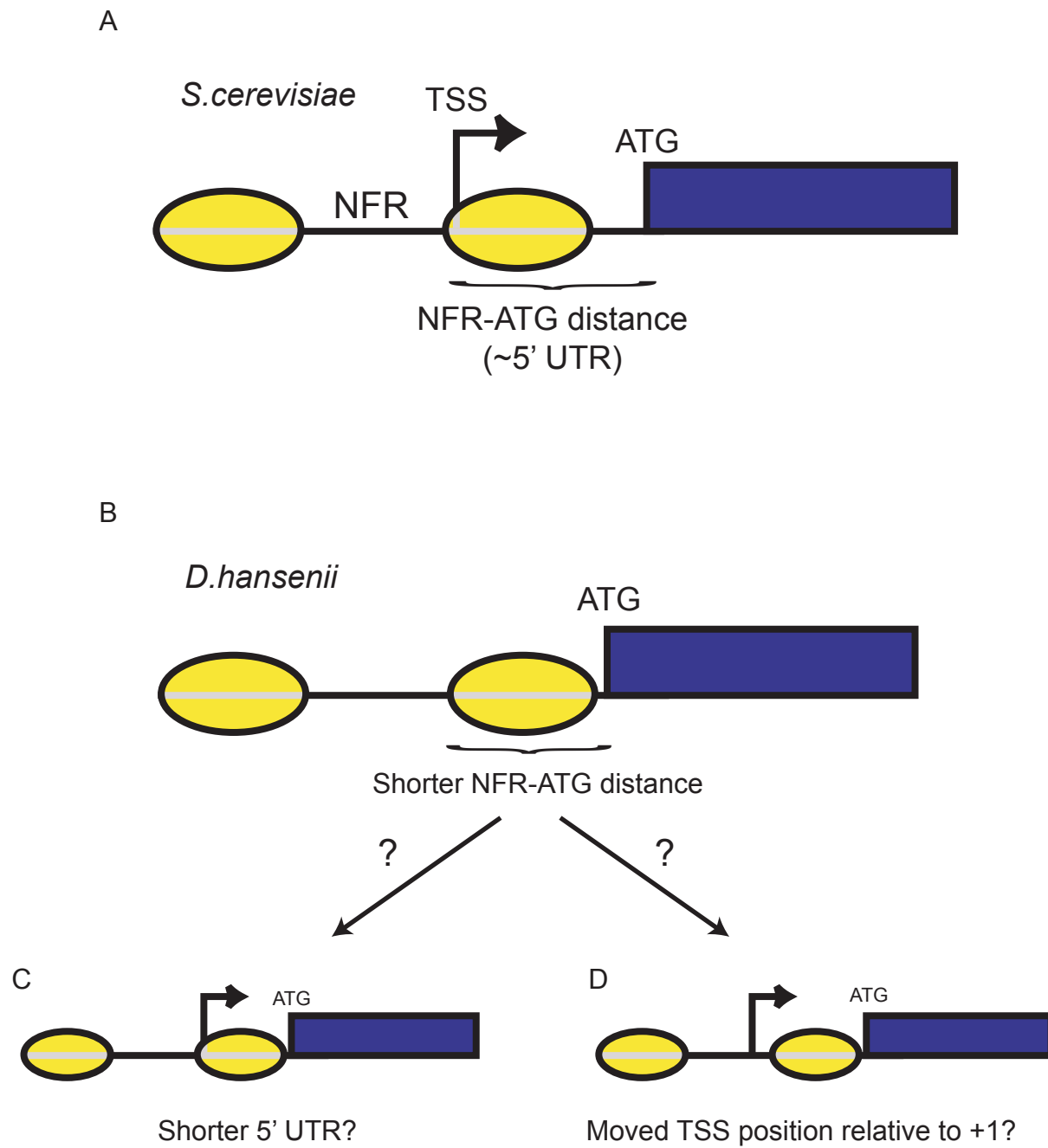

Supplement: Figure S5 — Two scenarios for changes in NFR-ATG distance. (A) Canonical promoter architecture in S. cerevisiae –transcriptional start site (TSS) is typically found at ∼13 nt 3′ to the upstream border of Nuc+1. (B) 5′NFR to ATG distance (D5′NFR-ATG) varies in other species without annotated TSSs. For example, NFR-ATG distance is shorter in D. hansenii than in S. cerevisiae (Figure 1E). Depending on the location of the TSS, this result is consistent with two possibilities (or any admixture thereof): (C) TSSs are located 13 nt into Nuc+1, and 5′ UTRs are globally shorter, or (D) 5′ UTRs are the same length and the TSS is situated within the NFR. Several lines of evidence support the latter possibility (D), including the conservation of 5′UTR length distribution in a small number of measured cases in S. cerevisiae and C. albicans [45], the known variation in TATA-TSS distances between S. pombe and S. cerevisiae [78], and the known variation between yeast, fly, and humans in TSS location relative to Nuc+1 [26],[46],[79],[80]. Thus, it is likely that TSS location relative to Nuc+1 varies substantially between Hemiascomycota species. This would affect TSS-exposure rates and pre-initiation complex geometry and has unknown consequences for basic gene regulatory mechanisms [16],[81]. (0.27 MB PDF) [file pbio.1000414.s005.pdf]

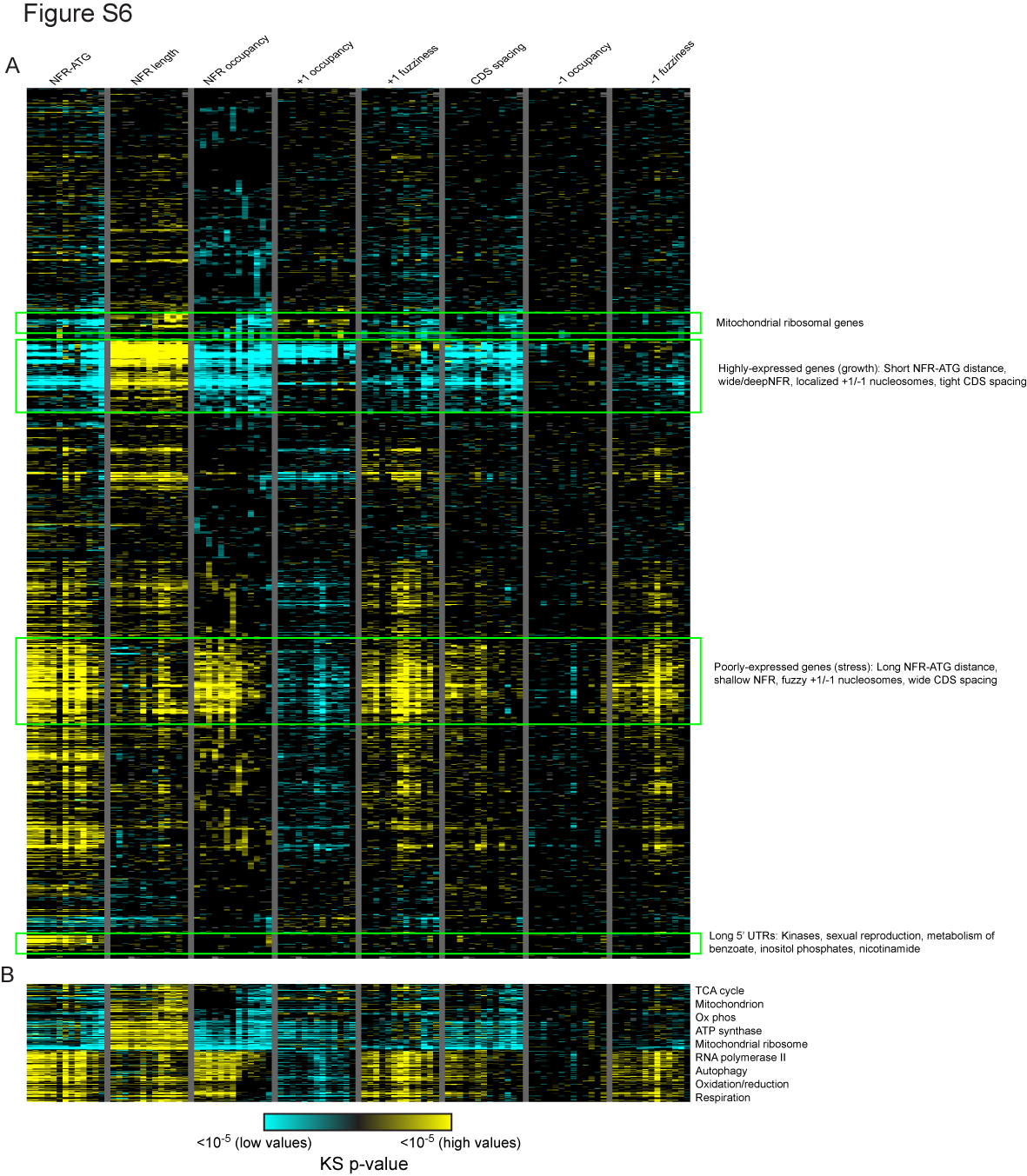

Supplement: Figure S6 — Conservation and variation in chromatin structure of functional gene sets. (A) Global overview of chromatin behavior within functional gene sets. K-S scores (Materials and Methods) were calculated for 8 parameters for 4,774 gene sets in each species as in Figure 2A,B. Only gene sets with over 10 members in 10 or more of species are shown (1,159 gene sets, including “transcriptional modules” and genes annotated based on expression changes in deletion strains [34], both excluded from Figure 2). Gene sets were clustered by K-S scores, and enrichments are shown as in Figure 2C–J. Selected clusters of gene sets are marked on the right. Note that stress-related gene sets tend to become less enriched for various chromatin and expression features at increasing phylogenetic distance from S. cerevisiae, likely due to the rapid gain/loss of these genes over this phylogenetic distance [34]. Importantly, genes in distant species associated with orthogroups lacking an S. cerevisiae member tend to be poorly expressed and exhibit stress-related chromatin characteristics (unpublished data), indicating that these genes likely play species-specific stress-related roles. (B) Gene sets associated with increase in NFR occupancy in post-WGD species were identified and are shown as in panel A. (0.61 MB PNG) [file pbio.1000414.s006.png]

Figure S7

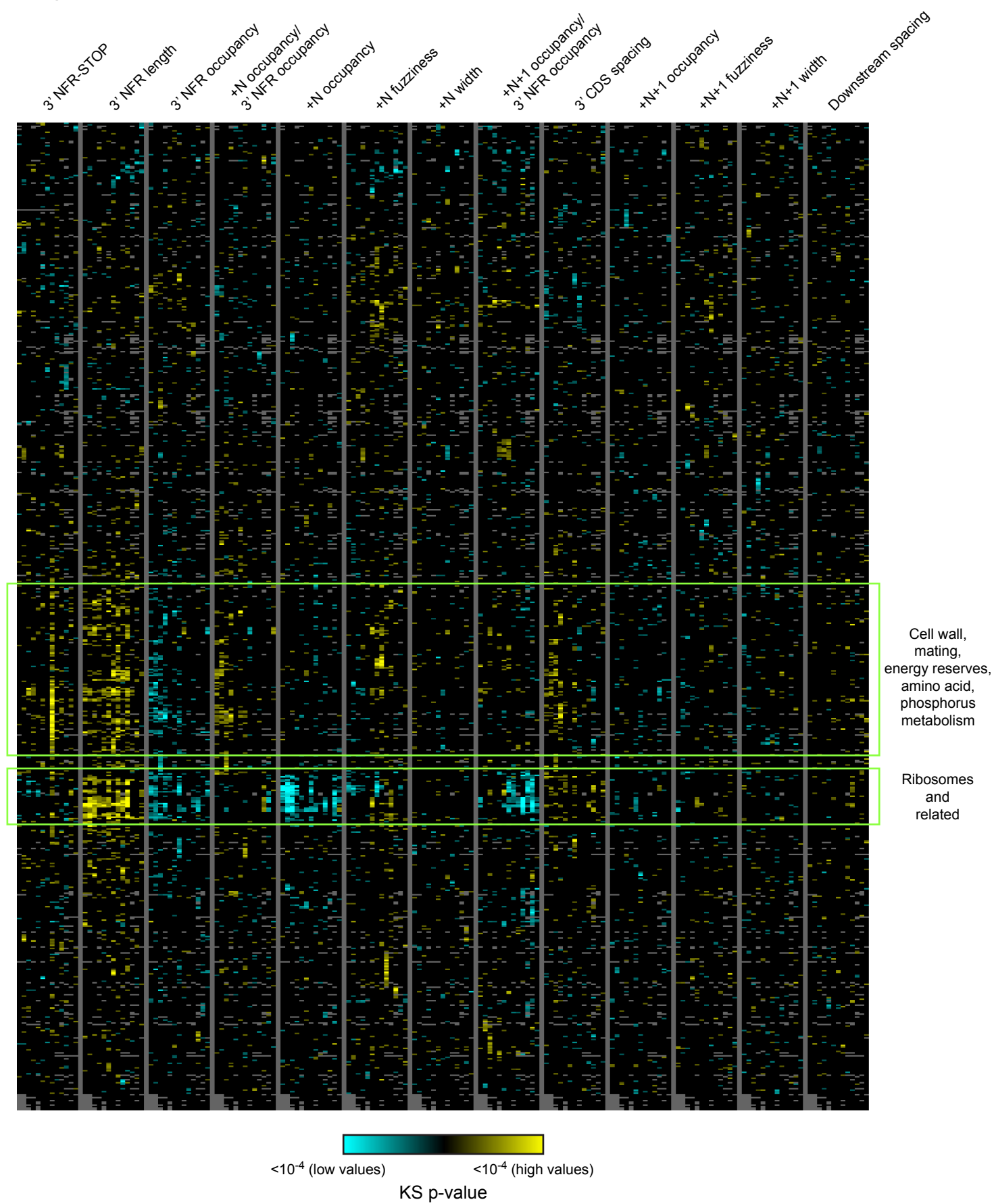

Supplement: Figure S7 — 3′ NFR enrichments. K-S enrichments for 3′ chromatin parameters were calculated for all gene sets as in Figure 2, considering only genes in convergent (tail to tail) orientation. K-S scores were clustered as in Figure S6. Few enrichments are apparent, most notably enrichments of long 3′ NFRs (and either low Nuc+N occupancy or tight 3′ CDS spacing) downstream of ribosomal protein genes. Additional annotations associated with long 3′ NFRs are noted on the right. (6.80 MB PDF) [file pbio.1000414.s007.pdf]

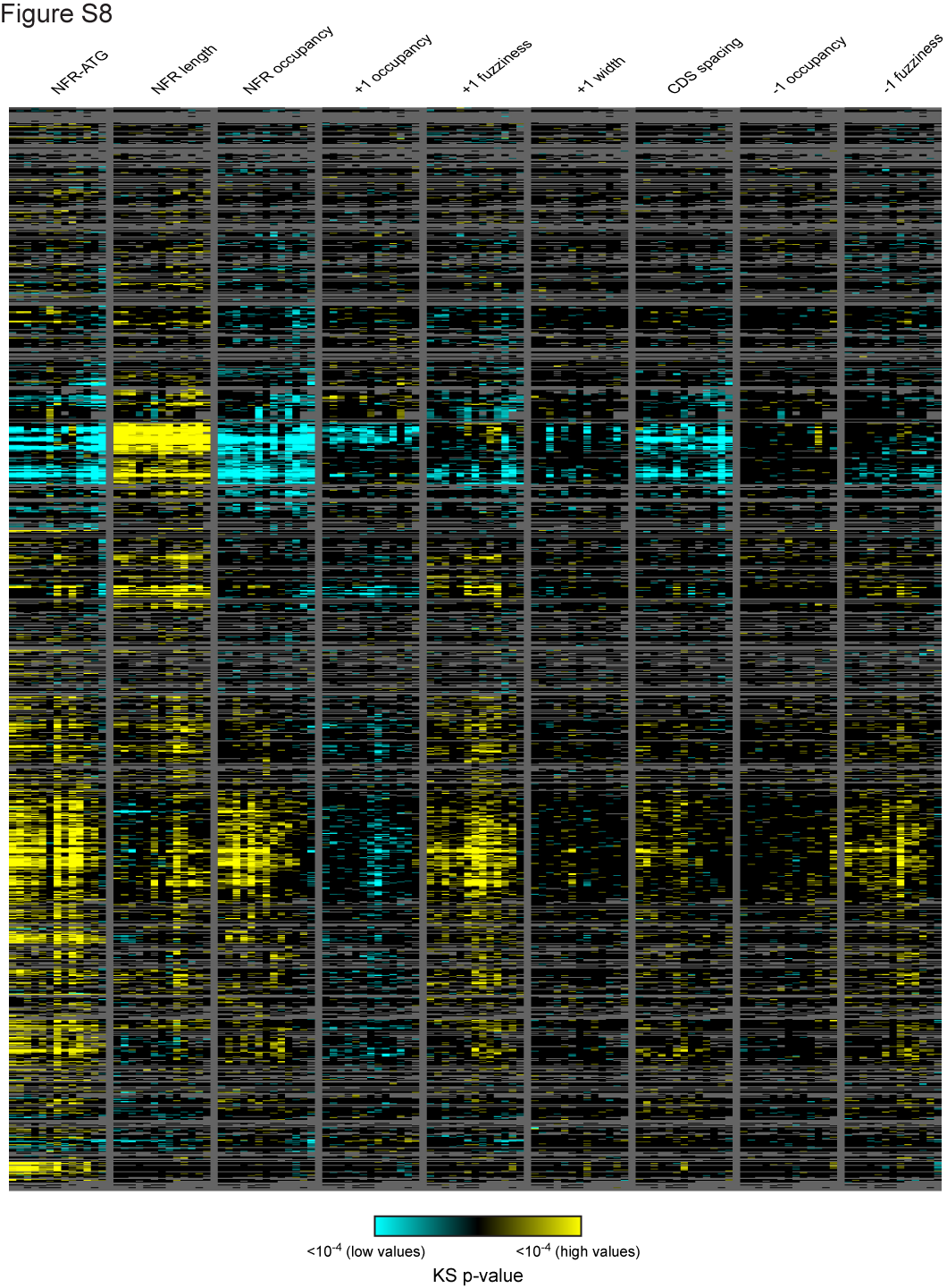

Supplement: Figure S8 — Chromatin feature enrichment for gene sets is robust to gene orientation. K-S enrichments for chromatin parameters were calculated for all gene sets as in Figure 2A,B and Figure S6, but only considering genes in tandem (head to tail) orientation. Annotations are ordered as in Figure S6. The increased number of grey boxes indicates gene sets with less than 10 members when divergently oriented promoters are excluded. (0.60 MB PNG) [file pbio.1000414.s008.png]

Figure S9

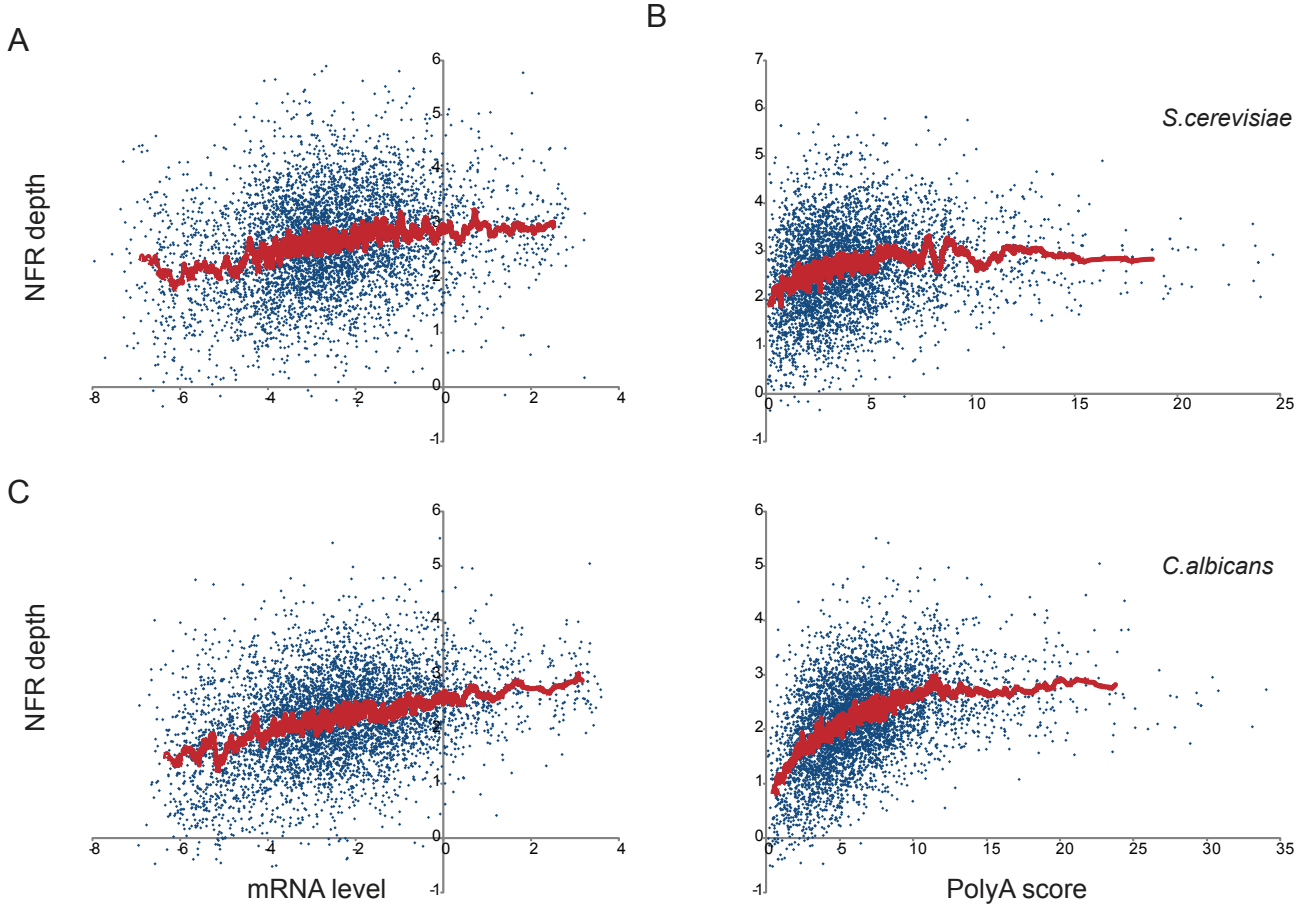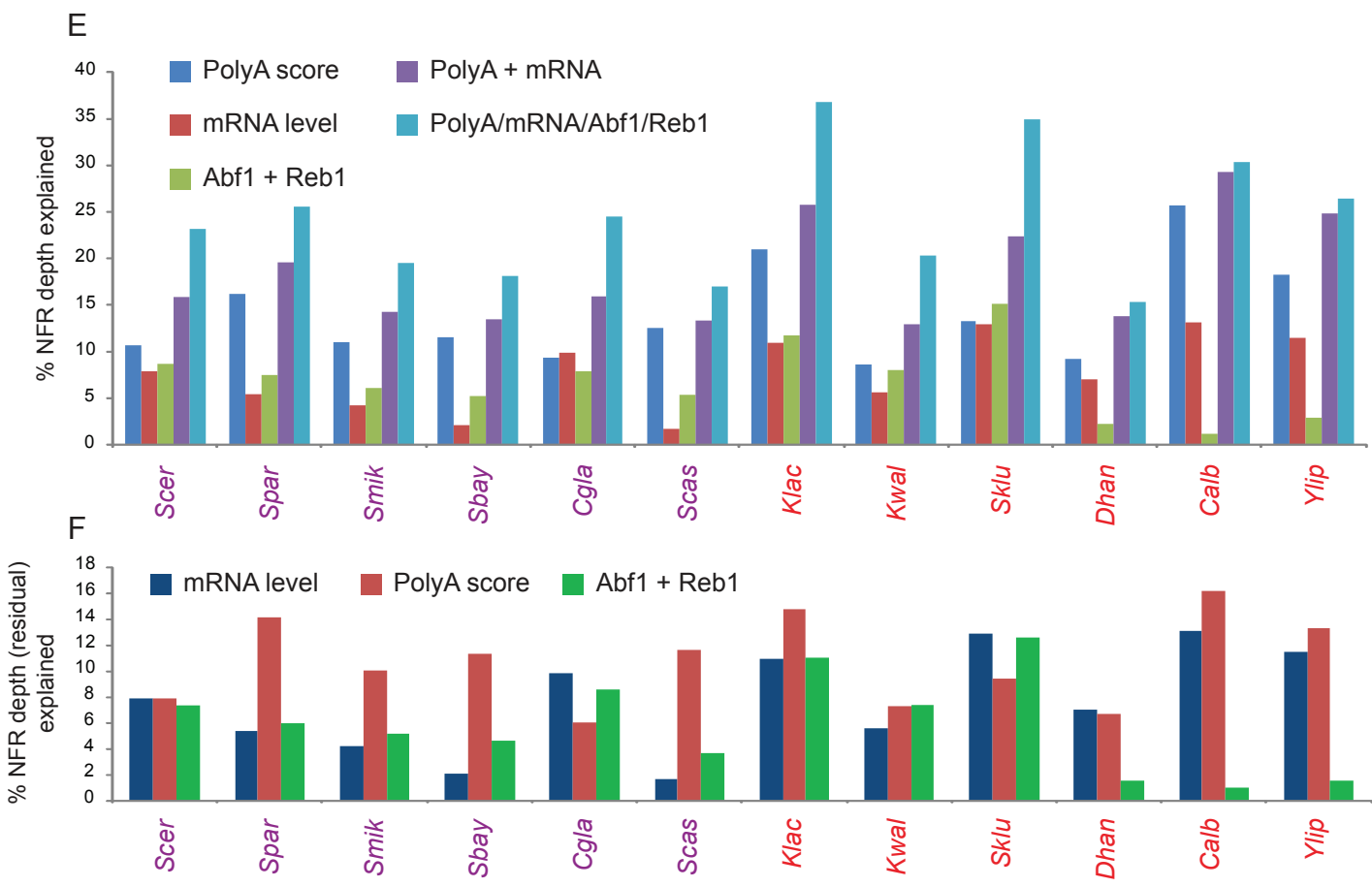

Supplement: Figure S9 — Relationship between RNA level, antinucleosomal tracts, GRF sites, and chromatin structure. (A–D) Gene-by-gene comparisons of NFR depth to mRNA levels or Poly(dA:dT) signal. Shown are plots of NFR depth (y-axis) versus mRNA level (A,C) or Poly(dA:dT) score at the NFR (B,D) for each gene (blue dot) in the S. cerevisiae (A,B) or C. albicans (C,D) genome. Also shown is a 50-gene running window average for each panel (red). (E) Variation in NFR depth explained by each determinant and their combination. Shown are the % variation in NFR depth (bars, y-axis) explained in each species by each determinant alone (dark blue, polyA; red, mRNA expression; green, binding sites for Abf1 and Reb1 in the NFR) and two combinations (purple, polyA and mRNA expression; light blue, polyA, mRNA, and the GRF sites). (F) The residual contribution of polyA and GRF sites. Shown are the % variation in NFR depth (bars, y-axis) explained in each species by mRNA expression alone (blue bars), the subsequent residual variation explained by polyA (red bars) and the residual variation (after mRNA and polyA) explained by the binding sites for the GRFs Abf1 and Reb1 (green). (2.97 MB PDF) [file pbio.1000414.s009.pdf]

Figure S10

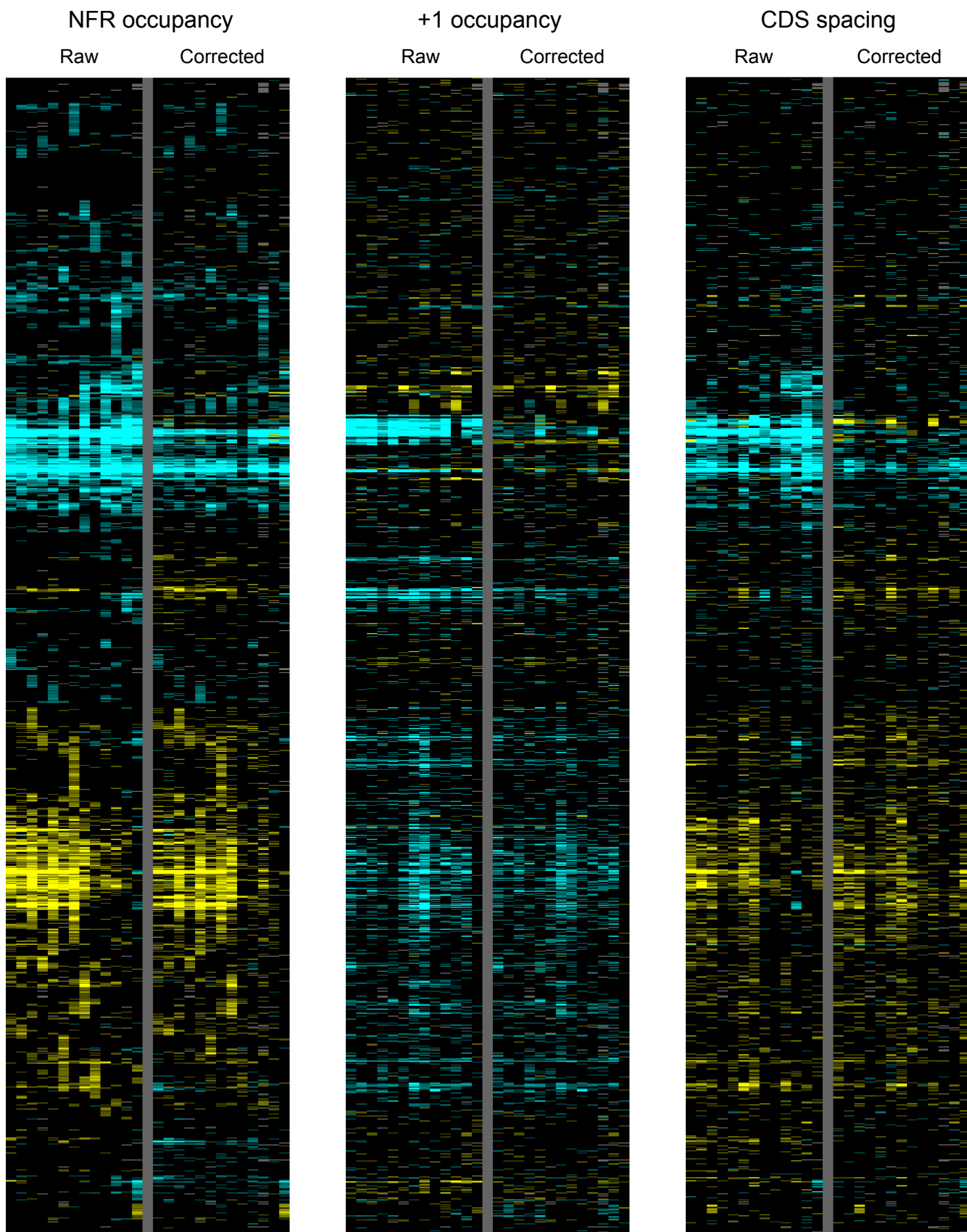

Supplement: Figure S10 — Relationship between RNA level and chromatin structure. The extent of variation in a given chromatin parameter which is explained by RNA abundance was calculated (LOWESS, Materials and Methods) for each feature in each species. The fitted LOWESS curve was then used to correct for the effect of transcription on chromatin packaging, and K-S enrichments were recalculated as in Figure 2A,B. Shown are K-S enrichments, as in Figure S6, for gene sets calculated before (“Raw”) and after (“Corrected”) LOWESS-correction. NFR occupancy enrichments are not strongly influenced by RNA levels, whereas Nuc+1 occupancy and CDS nucleosome spacing enrichments were more substantially explained by RNA abundance measures. (5.52 MB PDF) [file pbio.1000414.s010.pdf]

Figure S11

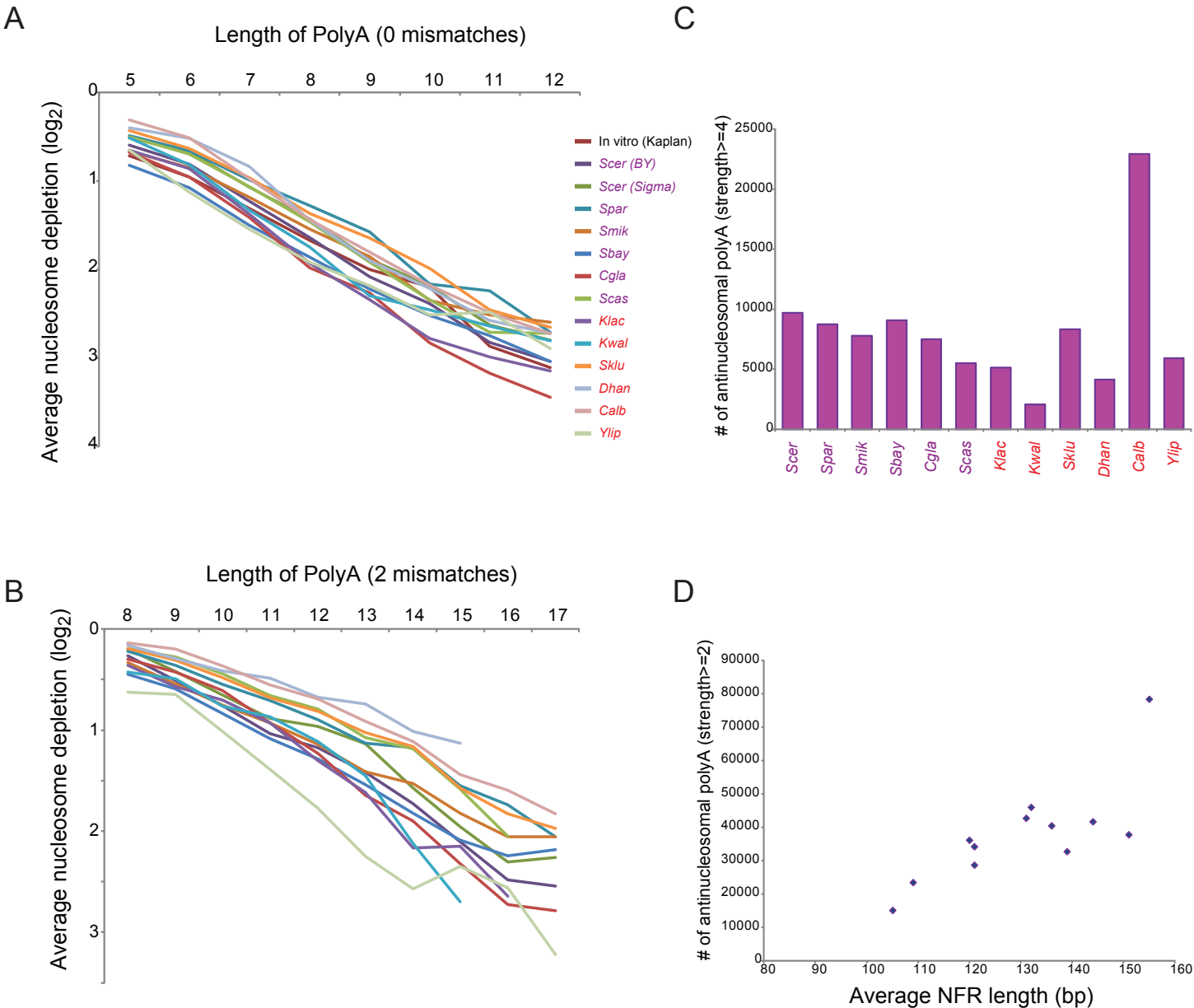

Supplement: Figure S11 — Relationship between Poly(dA:dT) tracts and nucleosome depletion varies between species. (A,B) Shown are plots of nucleosome depletion (log2, y-axis) versus length of Poly(dA:dT) tract (x-axis) for Poly(dA:dT) tracts with no mismatches (A) or 2 mismatches (B). (C) Species differ in the number of antinucleosomal PolyA tracts. Shown are the number of anti-nucleosomal Poly(dA:dT) tracts with a strength score greater than 4 in the NFRs of each species. (D) Median NFR width (per species) is correlated (r = 0.77) with number of anti-nucleosomal Poly(dA:dT) tracts in the NFR. Shown are the number of anti-nucleosomal Poly(dA:dT) tracts with a strength score greater than 2 in the NFRs in each species versus that species' average NFR length. (0.37 MB PDF) [file pbio.1000414.s011.pdf]

Figure S12

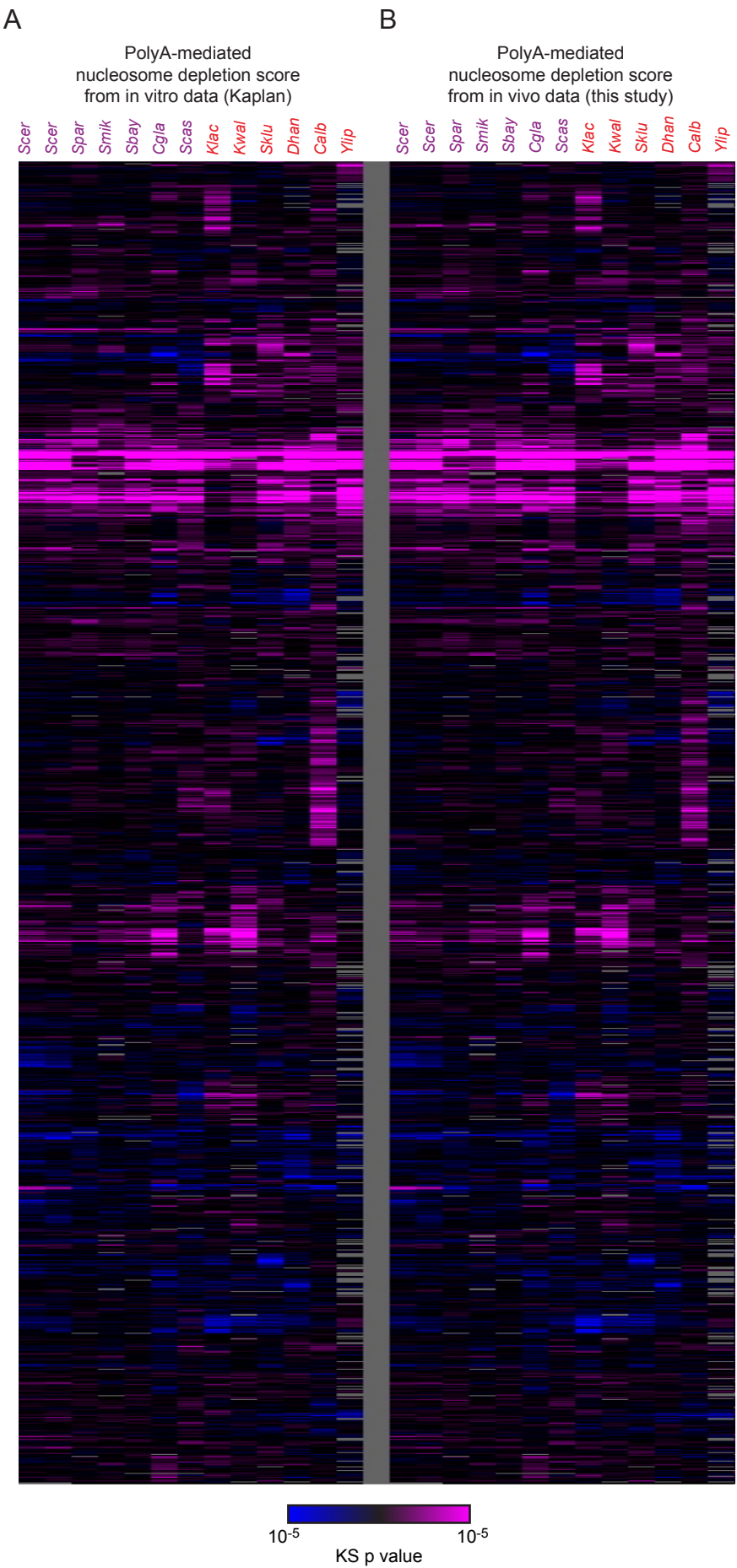

Supplement: Figure S12 — The calculated effect Poly(dA:dT) tracts on nucleosome depletion for gene sets is independent of the dataset used. We calculated the extent of nucleosome depletion over various lengths of Poly(dA:dT) using either in vitro nucleosome reconstitution data [21] (A) or our in vivo nucleosome mapping data from all studied species (B). Shown are K-S enrichments for gene sets ordered as in Figure S6. (4.39 MB PDF) [file pbio.1000414.s012.pdf]

Figure S13

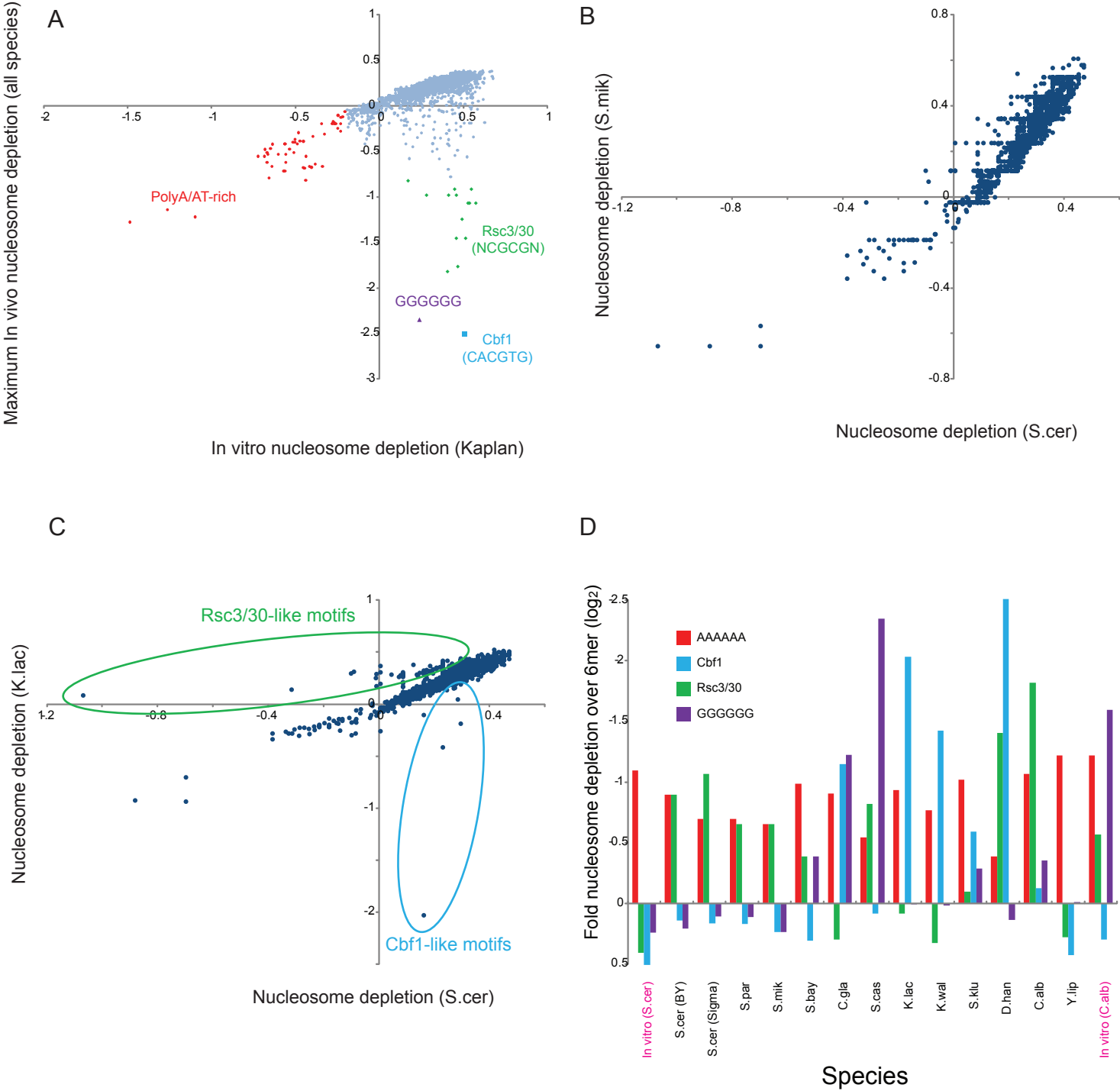

Supplement: Figure S13 — Analysis of anti-nucleosomal 6-mers. (A) The extent of nucleosome depletion over all 6-mers was calculated as in Figure 3A. Shown is a scatter plot of the nucleosome depletion observed in in vitro reconstitutions [21] versus the maximal nucleosome depletion observed in vivo in any of the 12 species in this study. (B) In vivo nucleosome depletion of each 6-mer in S. cerevisiae is plotted against that in S. mikatae. Few differences are observed. (C) As in (B), but for S. cerevisiae versus K. lactis. CGCG-containing Rsc3/30-like motifs (green) are more nucleosome-depleted in S. cerevisiae than in K. lactis, whereas the Cbf1 motif CACGTG and related motifs (blue) are more nucleosome-depleted in K. lactis than in S. cerevisiae. This is consistent with the loss of the Rsc3/30 ortholog in K. lactis [82]. (D) Nucleosome depletion score for four major anti-nucleosomal 6-mers across 13 in vivo datasets and 2 in vitro datasets [21],[32]. (0.72 MB PDF) [file pbio.1000414.s013.pdf]

Figure S14

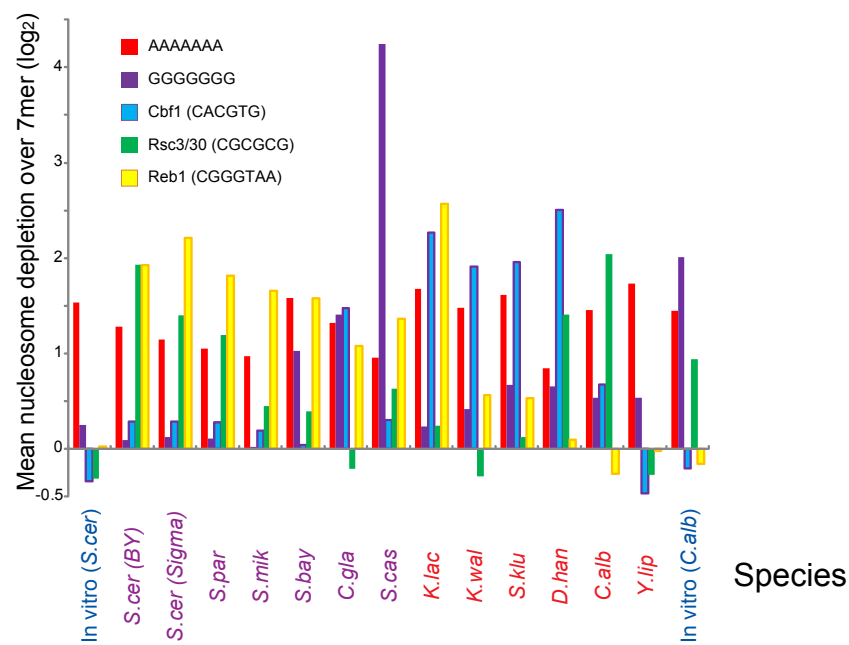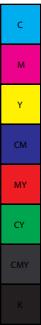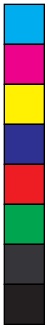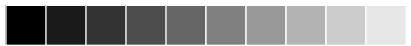

Supplement: Figure S14 — Species-specific usage of GRF-related motifs. Shown are nucleosome depletion scores over the indicated sequences for all in vivo data reported here, and for two published in vitro reconstitution datasets (blue) in S. cerevisiae [21] and C. albicans [32]. All shown elements are associated with nucleosome depletion in at least one species. (0.32 MB PDF) [file pbio.1000414.s014.pdf]

Figure S15

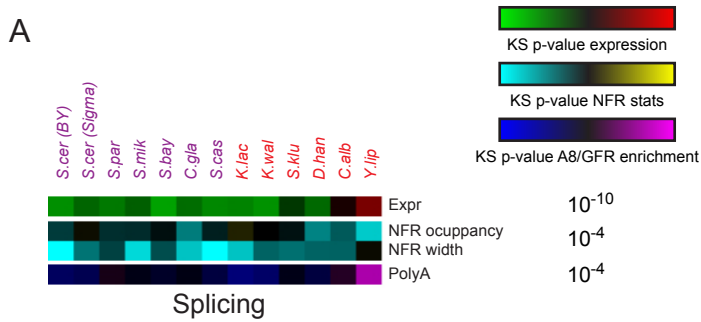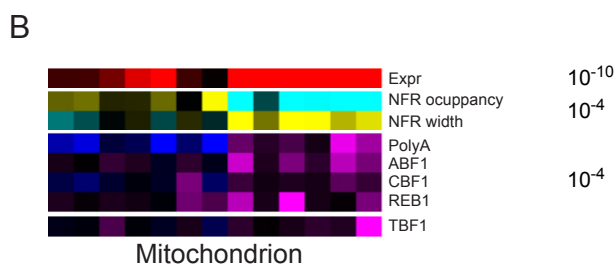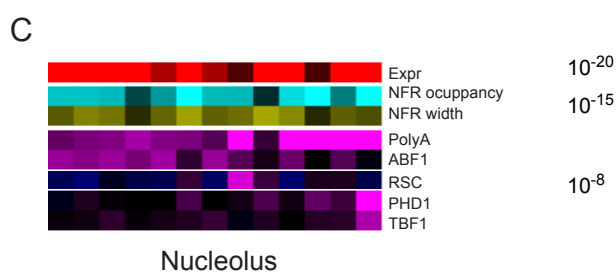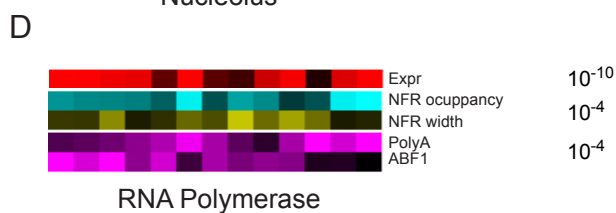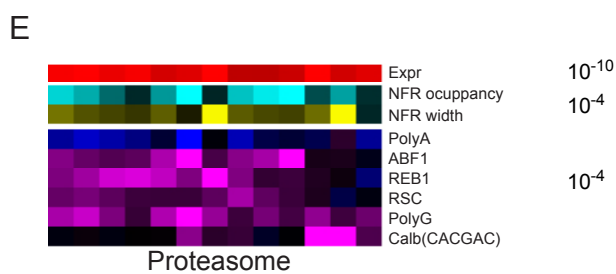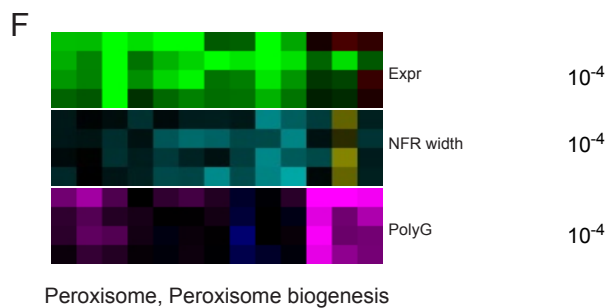

Supplement: Figure S15 — Evolution of anti-nucleosomal programming at specific gene sets. Enrichment of Poly(dA:dT) tracts (A8) or motifs for various GRFs was calculated for the indicated gene sets. Enrichments are shown for high (red) or low (green) expression levels, high (yellow) or low (blue) 5′NFR occupancy or length, and enrichment (pink) or depletion (blue) of A8 or GRF motifs for each gene set. K-S P value saturation levels are indicated to the right of each panel. (0.46 MB PDF) [file pbio.1000414.s015.pdf]

Figure S16

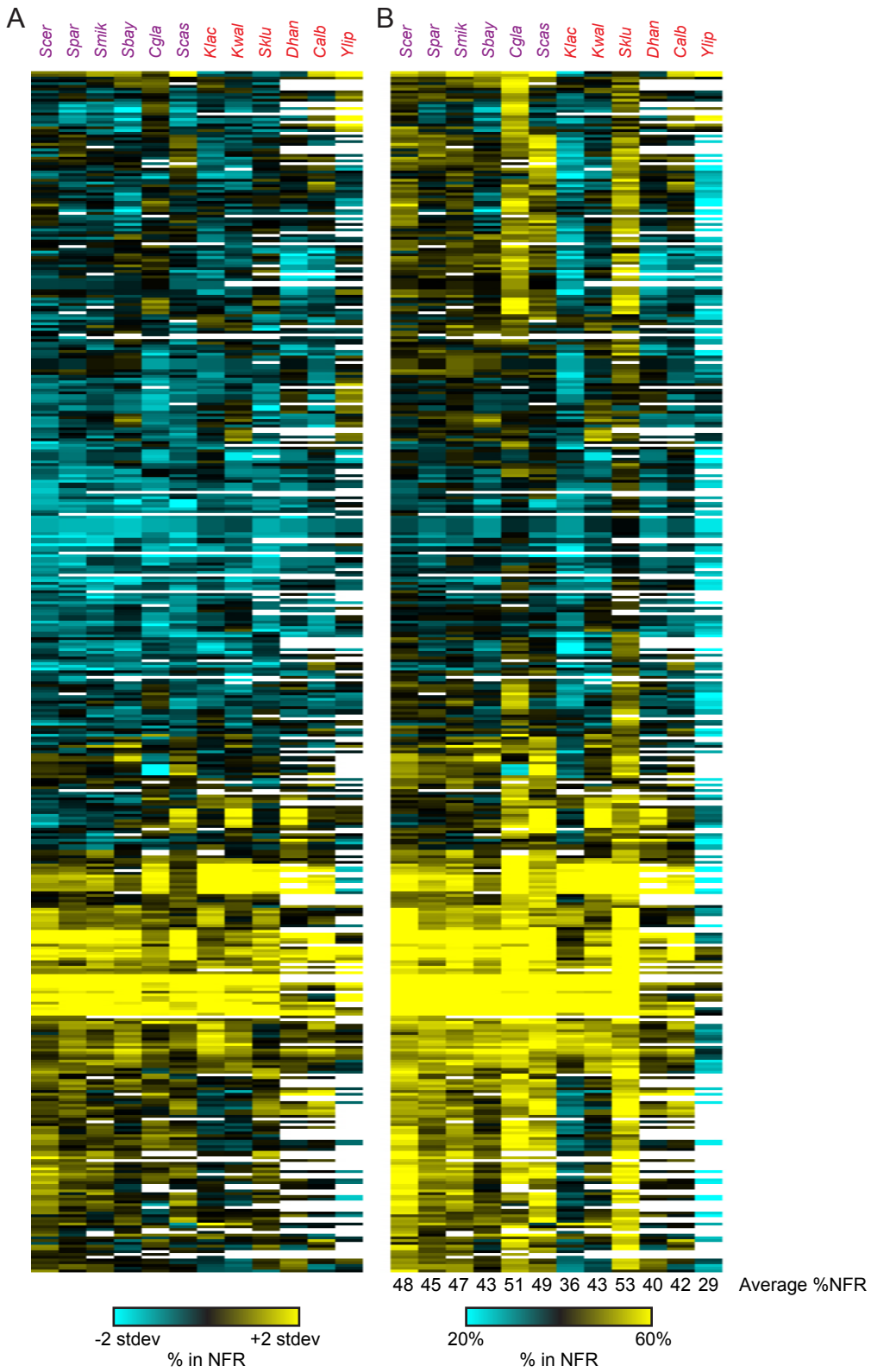

Supplement: Figure S16 — Re-positioning of TF motifs relative to NFRs. (A) Location of TF binding sites relative to NFRs in all 12 species (identical to Figure 4C): blue, NFR depleted; yellow, NFR enriched. Since the fraction of sites in NFRs varies with average NFR width and phylogenetic distance from S. cerevisiae, the fraction of motif instances located in NFR for each species was normalized by each species' mean and standard deviation. White, S. cerevisiae protein motifs whose orthologs are absent from a given species. (B) As in (A) and Figure 4C, but without scaling each species. The overall fraction of motifs located in NFRs decreases with increasing phylogenetic distance from S. cerevisiae, likely due to variation in TF binding site affinities. Much of the variation in the overall fraction of motifs in NFRs can also be ascribed to variation in NFR length across species—C. glabrata, for example, exhibits unusually long NFRs (Table S3), and the resulting high motif localization to NFRs is therefore corrected by normalization to overall % NFR in panel A and Figure 4C. (1.69 MB PDF) [file pbio.1000414.s016.pdf]

Figure S17

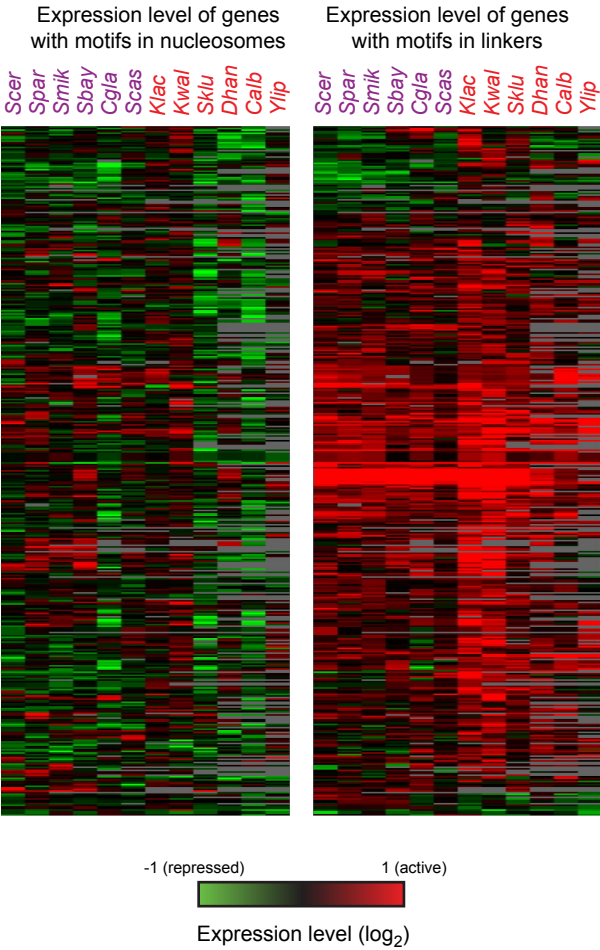

Supplement: Figure S17 — Divergence of activity of NFR-localized binding sites. Variation in TF activity across the 12 species. Data shown as in Figure 5A–B, but for all TF motifs, for all species studied (columns). (1.26 MB PDF) [file pbio.1000414.s017.pdf]
